# COVID-19 Vaccine Passport and International Traveling: The Combined Effect of Two Nudges on Americans’ Support for the Pass

**DOI:** 10.3390/ijerph18168800

**Published:** 2021-08-20

**Authors:** Chiara Sotis, Miriam Allena, Renny Reyes, Alessandro Romano

**Affiliations:** 1 Department of Geography and Environment, London School of Economics, London WC2A 2AE, UK; 2 Law School, Bocconi University, 20136 Milan, Italy; miriam.allena@unibocconi.it (M.A.); alessandroromano@unibocconi.it (A.R.); 3 Law School, Pontificia Universidad Católica Madre y Maestra, Santiago de los Caballeros 11403, Dominican Republic; rennyreyes@gmail.com; 4 Law School, Yale University, New Haven, CT 06520, USA

**Keywords:** COVID-19, COVID-19 vaccine, vaccine passport, nudges, interaction between nudges, peer effect, status quo bias

## Abstract

Immunity passports have the potential to allow large-scale international traveling to resume. However, they can only become an effective tool if they are widely supported by the general public. We carry out a double blind randomized online experiment with a sample of N=4000 Americans to study (*i*) whether two nudges can increase the level of support for a COVID pass for international traveling, (*ii*) the relationship between the effects of the nudges, and (*iii*) if these nudges have a negative spillover on the intention to get vaccinated. We find that both nudges increase the support for the COVID pass and that their impact is stronger when they are used together. Moreover, we find that the two nudges do not negatively affect intentions to get vaccinated. Our findings have important implications for policymakers and for the nascent literature on the interaction between multiple nudges.

## 1. Introduction

The coronavirus disease 2019 (COVID-19) pandemic has caused catastrophic losses both in terms of human lives [1] and to the economy [2,3]. Some of the sectors that were hit most dramatically are those connected with international travel. For instance, the tourism sector and the airline industry have been severely affected by the many traveling restrictions adopted worldwide [4] and by the obvious reluctance of many to travel during a pandemic [5]. According to the The World Travel and Tourism Council, in 2020, the travel and tourism sector experienced staggering losses of 3.8 trillion USD [6]. Moreover, a new report from the UN’s Air Transportation Agency indicates that COVID-19 caused a decline in international air travel of around 60%, which resulted in losses of over 370 billion USD for the airline industry [7]. However, as existing vaccines have been proven to be safe and effective [8,9], these key industries are looking forward to better times, especially in light of the possible introduction of COVID-19 vaccine passports (hereafter, COVID passes). COVID-19 passes allow the bearer to show information on their immunization status and permit people who have been vaccinated against COVID-19, or who have recently recovered from the virus, to face fewer restrictions while traveling.

While COVID passes present significant ethical and scientific challenges [10], requiring proof of vaccination for international travel is not a new practice. For instance, the World Health Organization (WHO) has long endorsed certificates confirming vaccination against yellow fever to travel to certain countries [11]. Building on this precedent, policymakers and private companies have either introduced, or are planning to introduce, some form of COVID pass for international travel. For example, the European Commission has reached an agreement on the “Digital COVID Certificate”, which will provide a proof that a person has been vaccinated against COVID-19, received a negative test result, or recently recovered from COVID-19 [12]. Moreover, leading airlines are working on the IATA Travel Pass App and are considering allowing only holders of such a pass to board their flights [13].

In order to be successful, COVID passes need to be widely supported by the public. From this perspective, the demise of Contact Tracing Apps is a case in point [14] showing that even the most promising and well-regarded technological innovation can fail to deliver if it is perceived negatively by potential adopters. In a similar vein, if COVID passes are implemented without the support of the general public, they might lead to significant problems such as reducing vaccine uptake [15]. Luckily, at least for international travel, COVID passes seem to be perceived somewhat favorably among the general public. A recent survey carried out in the U.S. reveals that only about one third of Americans is against requiring proof of vaccination for international travel in the form of a COVID pass [16], while in a study carried out by IATA, 80% of respondents stated that they intend to use the IATA Travel Pass App as soon as it becomes available [17].

Due to their enormous potential impact, COVID passes have been hotly discussed in the academic literature [18,19,20,21]. However, all existing studies have been purely qualitative or observational [22], with one notable exception [23]. Recent research related to COVID-19 has shown that behavioral interventions can have a significant impact on people’s perception of the pandemic and foster people’s preventive and pro-social behaviors [24,25,26,27,28]. The literature has shown that experimental studies can offer precious guidance to policymakers and companies. This paper expands this literature and offers guidance on how to increase the support for a COVID pass through a communication campaign based on nudging.

In particular, we present the results from a double blind randomized online experiment with a sample of N=4000 Americans to test (*i*) whether two nudges can increase the level of support for a COVID pass for international traveling, (*ii*) if there are synergies between the effects of the two nudges, and (*iii*) whether these nudges generate negative spillovers on intentions to get vaccinated.

The first nudge exploits the status quo bias, which is an effective technique to increase the acceptance of a policy by presenting it as a sign of continuity with the past and has proven effective in a variety of contexts [29]. Here, we note that proof of vaccination for international travel is not a novel idea and hypothesize that this will increase the support for the COVID pass. The second nudge instead builds on the peer effect. There is evidence that information on a person’s peers’ actions can induce pro-social behaviors [30,31] and that people tend to conform to the policy preferences of their peers [32]. Moreover, previous literature has shown a tendency to conform driven by a need to belong to a group and the influence that the group’s opinion has on the individual [33,34]. In this vein, we hypothesize that informing respondents about the limited opposition to COVID passes for international travel would increase the support for this policy.

## 2. Background Literature and Theoretical Framework

Nudges are now widely accepted as an effective tool to influence behaviors without constraining individuals’ ability to choose [35,36,37]. Recent studies have shown that nudges are effective even in the context of COVID to promote pro-social behaviors [25,26,27]. Here, we investigate whether they can also be used to increase the support for the COVID pass.

### 2.1. Status Quo Bias

The first nudge on which we rely is the status quo bias. The basic idea is that people are more likely to support a policy if it is perceived as a continuation of the past. In other words, whenever “an advertiser, political actor, or any other persuader wishes to make a practice or product acceptable, framing their preferred alternative as the status quo is likely to enhance its position and increase its support” [38]. For instance, a recent study shows that support for carbon mitigation policies is higher if they are presented as a continuation of the status quo [39]. Similar results are obtained even for practices as controversial as torture. Crandall et al. [38] observe that when torture is presented as a longstanding practice, it is perceived as more justifiable and effective. Scholars have advanced a wide array of explanations for the existence of this bias ranging from loss aversion and regret avoidance to repeated exposure [40]. However, there is evidence that the bias also stems from people’s assumption of goodness associated with the mere existence and longevity of a given state of the world [40]. Against this background, it is reasonable to expect that the status quo bias can be exploited in connection with the COVID pass. In particular, highlighting that requiring proof of vaccination for international travel is not unprecedented should trigger the status quo bias and hence increase the support for the COVID pass.

### 2.2. Peer Effect

The second nudge we test is peer effect. Several studies found that social norms and peer influence can shape behaviors and attitudes [30,31]. In a similar vein, other studies find that poll results can influence individual-level attitudes, triggering the so-called “bandwagon effect” [32]. When polls indicate that a policy is widely supported, even more people will be persuaded to support that policy [41]. Thus, polls not only describe public opinion, but can also influence it. In fact, in many democratic countries, there are restrictions on carrying out and publishing polls before elections [42]. In the context of policy support, the bandwagon effect causes an increase in support for a given policy motivated by the popularity of the policy itself. As the COVID pass for international travel already shows a relatively good level of support among segments of the public, we attempt to leverage the bandwagon effect to further increase the support for the pass.

### 2.3. Interaction among Nudges

Policymakers are increasingly relying on nudges to promote certain behaviors. Therefore, a key question is how multiple nudges targeted at promoting a given behavior interact. Consider the case in which nudges A and B are both effective in fostering behavior X; what happens when they are used simultaneously on the same target behavior?

**Figure 1:**
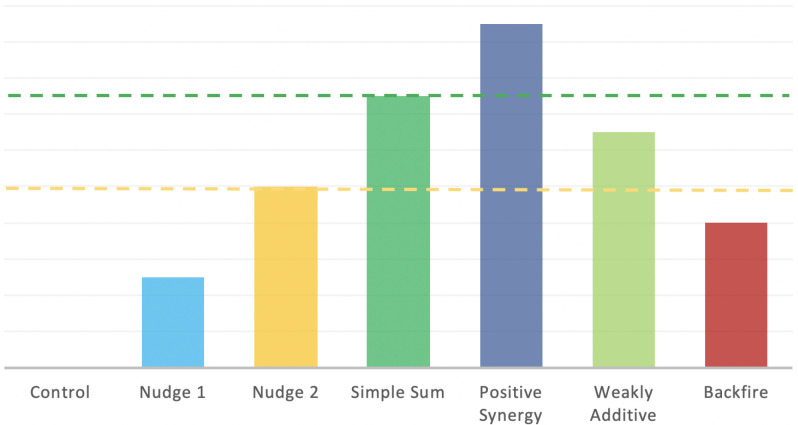
Possible interactions among nudges.

As indicated in Figure 1, the interaction between two nudges can (*i*) be synergistic, when their joint effect is larger than the sum of the effects of each nudge separately; (*ii*) be weakly additive, when their joint effect is larger than the effect of each of the two nudges when used separately, but smaller than their sum; or (*iii*) backfire, when the two nudges together produce a smaller effect than either of the two nudges used alone. Understanding the kind of interaction between nudges is extremely important, as policymakers generally employ a portfolio of tools and nudges to achieve a given goal [43]. The evidence produced by the literature is, however, very limited. A recent study focused on the interaction between a moral suasion nudge aimed at reducing electricity consumption during peak load events and peer effect comparison targeting aggregate household electricity consumption [44]; the study identified a synergistic relationship between the two nudges.

A related nascent area of literature has attempted to identify the relationship between price incentives and nudges [43]. The handful of works carried out in this domain have produced conflicting evidence [45,46,47]. A recent survey on the issue concluded that, due to the very small number of works on this area, “our understanding... is limited, as studies rarely use an approach that allows properly assessing synergy or the causes and mechanisms of it” [43]. One hypothesis that has been advanced is that there might be diminishing marginal returns from policy pressure, and therefore the relationship among nudges might depend on the level of support for a given policy [43,44]. Thus, for example, two nudges might be in a synergistic relationship when a policy has a low level of support, but this may change to a weakly additive interaction when the support for the policy grows beyond a certain threshold.

We attempt to contribute to this nascent and important field of research on the interactions of nudges by investigating the relationship between two widely adopted nudges in a highly salient domain.

### 2.4. Behavioral Spillovers

Another important question is whether a nudge has significant spillovers that affect other activities; that is, if nudge A, which is intended to promote behavior *X*, also affects behavior *Y*. The literature has found evidence of both positive and negative spillovers in different domains. For instance, a study found a strong interdependence between fuel-efficient driving styles and willingness to reduce meat consumption [48]. One reason behind positive spillovers of this kind might be that people wish to perceive themselves (and to be perceived) as consistent and hence attempt to act in a consistent manner across different domains. In this vein, if they engage in pro-environmental behavior *X*, they are more likely to engage in pro-environmental behavior *Y* [49]. Instead, in other cases, scholars have observed negative spillovers. For example, a study observed that owners of electric cars felt less compelled to engage in pro-environmental behavior than owners of traditional cars [50]. One of the possible causes of negative spillovers is the so-called moral-licensing effect. If people feel that they have done their part, they are more likely to engage in negative behaviors.

Understanding the signs of spillovers is crucial because, in the presence of negative spillovers, nudges that appear to be effective might backfire by triggering a negative response on other behaviors. Scholars are therefore trying to devise experiments that investigate the existence of such spillovers and their signs. However, the debate is still ongoing, and most studies so far have focused on pro-environmental behaviors. Understanding whether there are spillovers from the introduction of COVID passes and their direction should be a key priority for policymakers. For instance, if promoting the COVID pass results in lower vaccination uptake, governments should be very careful before implementing this tool.

## 3. Materials and Methods

We recruited a sample of N=4000 Americans on Prolific.co. To be eligible, people had to be at least 18 years of age and be resident in the U.S.. Participants took on average approximately 5 min and a half to complete the survey, and they were paid 0.55 USD. The data collection started and finished on the 15 May 2021. We obtained informed consent from all participants prior to the beginning of the online survey, which was approved by the faculty ethics committees of Yale University, Bocconi University, and the London School of Economics.

To begin with, we asked respondents about their vaccination status, which allowed us to distinguish between respondents who completed their vaccination schedule and those who were still waiting for their first or second dose. Moreover, we asked respondents who had not yet completed their cycle whether they intended to complete their cycle or not. In the same vein, we asked respondents who had not received a vaccine whether they intended to get vaccinated when they had the opportunity.

**Figure 2:**
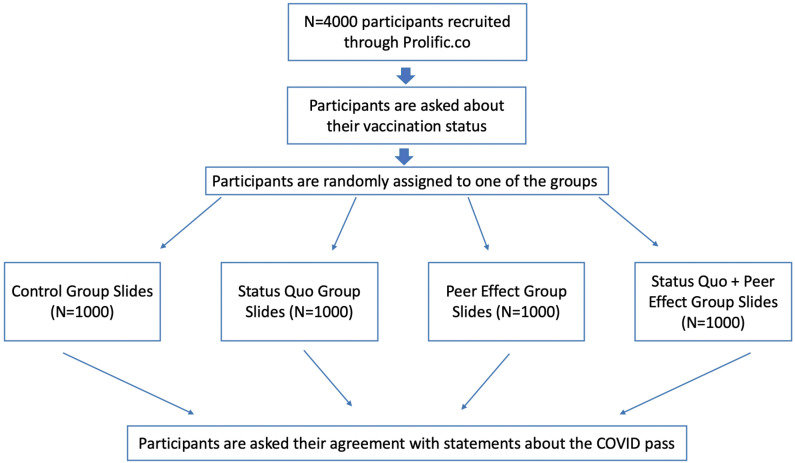
Experiment flow.

After this, respondents were randomly assigned to one of four different groups: Control, Status Quo, Peer Effect and Status Quo + Peer Effect (Figure 2).

**Figure 3:**
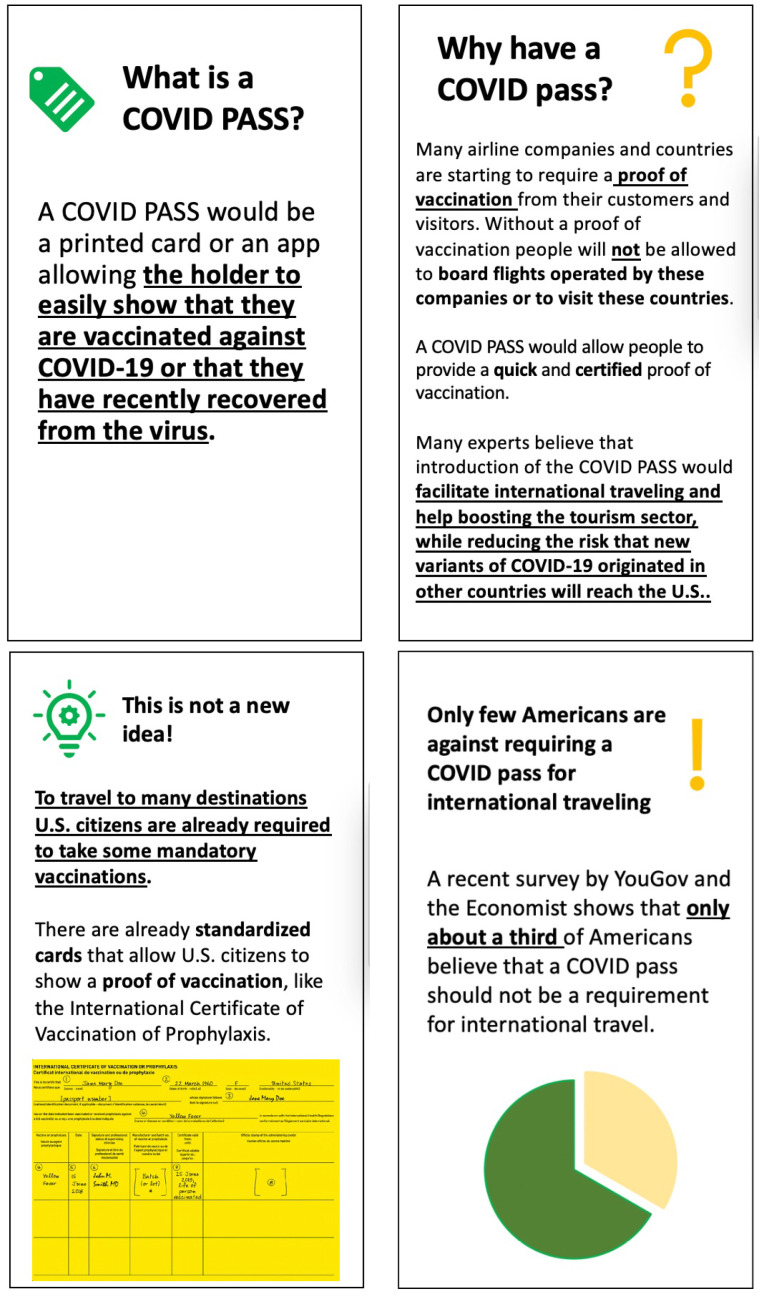
The top slides were shown to all participants. Respondents in the Control Group only saw the top slides. Respondents in the Status Quo Group also saw the bottom left slide. Respondents in the Peer Effect Group also saw the bottom right slide. Respondents in the Status Quo + Peer Effect Group saw all four slides.

The respondents in the Control Group only received basic information on the features and the purpose of a COVID pass for international travel. The respondents included in the Status Quo condition were also informed that requiring proof of vaccination for international travel is not unprecedented. Moreover, they were shown a picture of the International Certificate of Vaccination or Prophylaxis, or more simply the Yellow Card, endorsed by the World Health Organization to allow travelers to show proof of vaccination against yellow fever when entering certain countries. Finally, respondents in the Peer Effect condition were informed that according to a recent survey by YouGov and the Economist, only one third of Americans oppose a COVID pass for international traveling. Last, respondents in the Status Quo + Peer Effect condition were informed about both the fact that requiring proof of vaccination is not a novel idea and that only one third of Americans oppose a COVID pass for international travel (see Figure 3).

After seeing the treatment, respondents were asked to state their level of agreement on a scale from 0 to 10 with statements intended to capture their support for the COVID pass. The first 3 statements aimed at capturing the perceived importance of the COVID pass; statements 4–8 were designed to capture the perceived unfairness of the COVID pass in various dimensions; statements 9 and 10 targeted additional concerns connected with the COVID pass, namely whether it could be forged and how it would affect vaccination rates; statement 11 asked respondents whether they thought that only people with a COVID pass should be allowed to board international flights; statement 12 dealt with the troublesome finding of the literature that some people might intentionally become infected with COVID-19 if a COVID pass is introduced [51]; and statement 13 was designed to capture the overall perceived balance of the positive and negative aspects of the COVID pass. Table 1 reports the full list of statements, their number, and the summary statistics (mean and standard deviation). The number of the statements conveys the order in which they were asked and is used to denote the statements throughout the article.

Moreover, we asked respondents how likely they were to get vaccinated if a COVID pass for international travel was introduced. The precise wording of the question depended on the vaccination status declared by the respondents at the beginning of the survey. Therefore, for instance, respondents who had received only one dose were asked if they wanted to complete their vaccination cycle if the COVID pass were introduced. Instead, respondents who had not received a dose were asked if they intended to get vaccinated if a COVID pass were introduced. The answers are presented on a five-point Likert scale ranging from “very unlikely” to “very likely”.

Lastly, we asked a series of questions that were used as controls. These questions captured the level of trust in key institutions such as the Federal Government, the respondent’s State Government, the Centers for Disease Control and Prevention (CDC), or pharmaceutical companies and tech giants. Respondents also faced an attention test towards the end of the questionnaire. In the attention test, respondents received a multiple choice question in which they were asked to select the answer “5” if they were paying attention. The results presented in the next section exclude from the analysis the five participants who did not pass the attention test. However, the results remain robust even including these participants. We concluded the survey by asking standard demographic questions such as a respondent’s age, education, political affiliation, etc.

## 4. Results

In this section, we present the results of our experiment for each statement individually. In supplementary materials (Table S1), we present the results in a more concise form using factor analysis, grouping all the statements capturing the respondents’ support for the pass. The two approaches produce consistent results.

The demographics of our sample are similar to U.S. demographics in many key aspects (Table 2). For instance, roughly 76% of the U.S. population is white [52], and in our sample, white respondents account for 74% of the participants. Our sample also closely matches the general U.S. population with respect to income distribution, percentage of Republicans and education levels [52]. However, there are some differences in the composition of our sample and that of the US population. For example, 58% of our sample is composed of females, whereas the percentage of females in US is about 51% [53]. Our sample is also younger than the general population, but well represented in all age groups. Most importantly, the sample is balanced among conditions, allowing for a clear comparison between the control and treatment groups.

### 4.1. Status Quo Treatment

We start by studying the impact of the Status Quo treatment (Table 3). We find that respondents included in this group agree more with the three statements capturing the importance of the pass with respect to respondents included in the control group. The difference is sizeable, significant at 1% (p<0.001,p=0.002,p<0.001, respectively), and robust to different sets of control variables for all three statements. Regarding unfairness, we find that respondents in the Status Quo agree less with statements 4–7 (p<0.001, p=0.014, p=0.004, p=0.008). In this case, the difference is also sizeable and robust to a battery of controls. However, we do not observe a significant difference with respect to statement 8.

Additionally, we do not observe a significant impact of the Status Quo treatment on statements 9 and 12. Therefore, the Status Quo treatment does not induce people to believe that it is easy to forge a COVID pass, nor does it induce people to state that they would intentionally get COVID if a pass were introduced. Similarly, the Status Quo does not lead respondents to state that more people will get vaccinated if a pass were introduced (statement 10). Additionally, with respect to statement 11, we observe that the Status Quo leads respondents to agree more with the idea that only people with a COVID pass should be able to board international flights (p=0.024).

Lastly, respondents in the Status Quo condition agree more with statement 13 (p<0.001) and therefore consider the positives of a COVID pass to outweigh its negatives. Table 3 presents the results of regressions performed with the statement as a dependent variable, the treatment as the main independent variable, and controls for the demographics, vaccination status, and trust levels of the participants. We refer the reader to supplementary materials (Tables S2–S14) for the full regression tables, including the results from regressions with different sets of controls.

To summarize, we conclude that the status quo bias is highly effective in increasing the perceived importance of the COVID pass (statements 1–3), in reducing its perceived unfairness (statements 4–7, with the exception of statement 8), and in increasing the support for the COVID pass overall (statement 13).

### 4.2. Peer Effect Treatment

As for the Status Quo group, we find that respondents included in the Peer Effect group agree more with the three statements capturing the importance of the pass with respect to respondents included in the Control group (see Table 3). The magnitude of the effects is large, statistically significant (p<0.001 for all statements), and robust to various sets of control variables for all three statements. Considering unfairness, instead, we observe that the Peer Effect treatment is not effective. The Peer Effect treatment does not impact the level of agreement with statement 9 and 12, and it does not lead respondents to state that more people will get vaccinated if a pass were introduced (statement 10). Lastly, respondents in the Peer Effect group agree more with the idea that the the positives of a COVID pass outweigh the negatives (statement 13), but the result is weaker than for the Status Quo group (p=0.55). Table 3 presents the results of regressions performed with the statement as a dependent variable, the treatments as the main independent variable, and controls for the demographics, vaccination status, frequency of travel, and trust levels of the participants. We refer the reader to supplementary materials (Tables S2–S14) for the full regression tables, including the results from regressions with different sets of controls.

Overall, the peer effect condition has a positive and statistically significant impact on the perceived importance of the COVID pass (statements 1–3), but it has a limited impact on the perceived unfairness (statements 4, 6, 7, and 8, with statement 5 being the exception). Moreover, it increases the overall support for the COVID pass (statement 13).

### 4.3. Status Quo + Peer Effect Treatment

Then, we turn to the impact of the two nudges when they are used simultaneously. Starting with the statements on the importance of the pass, we find that the joint impact of the nudges is statistically significant (p<0.001 for all statements) and larger than the impact of both nudges used separately (Table 3), showing weak additionality in the treatments’ effects. For instance, for Statement 2, the joint impact of the two nudges is 218% larger than the effect of the Status Quo alone and and 162% larger than that of the Peer Effect.

Combining the two nudges is also very effective in reducing the perceived level of unfairness. Respondents in this condition agree less with statements 4, 5, 6, and 8 (p<0.001, p<0.001, p<0.001, p=0.059). In line with the Status Quo and the Peer Effect conditions, for statements 9, 10, and 12, we observe no significant differences between this condition and the Control. Instead, we see that for statement 11, the joint impact of the two treatments is slightly larger and more statistically significant (p=0.008) than that of the Status Quo and the Peer Effect when used separately.

Lastly, this treatment is the most effective in persuading people that the positives of the COVID pass outweigh the negatives (statement 13) (p<0.001).

Table 3 presents the results of regressions ran with the statement as a dependent variable, the treatment as the main independent variable and controls for demographics, vaccination status, frequency of travel and trust levels of the participants. We refer the reader to supplementary materials (Tables S2 – S14) for the full regression tables, including the results from regressions with different sets of controls.

**Figure 4:**
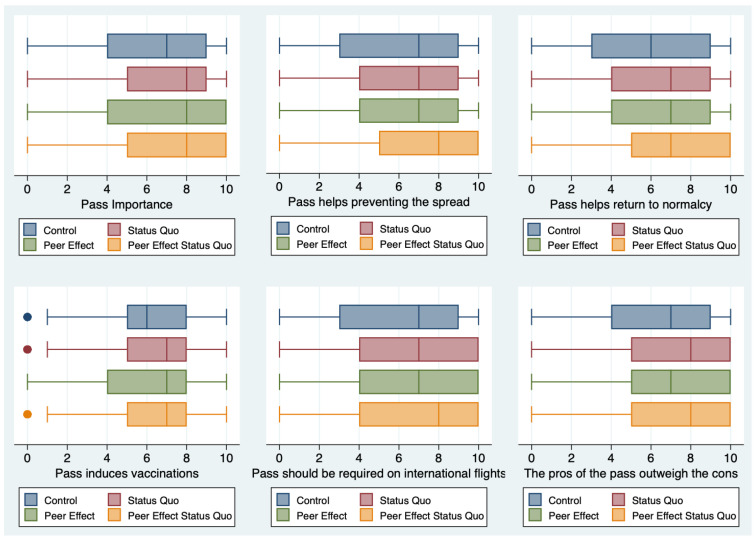
Summary of results for the statements meant to capture positive attributes of the COVID pass (statements 1–3, 10, 11, and 13). The boxplots show the distribution of responses in the Control, Status Quo, Peer Effect, and Peer Effect + Status Quo groups without any additional control. A higher value corresponds to more favorable attitudes towards the pass.

**Figure 5:**
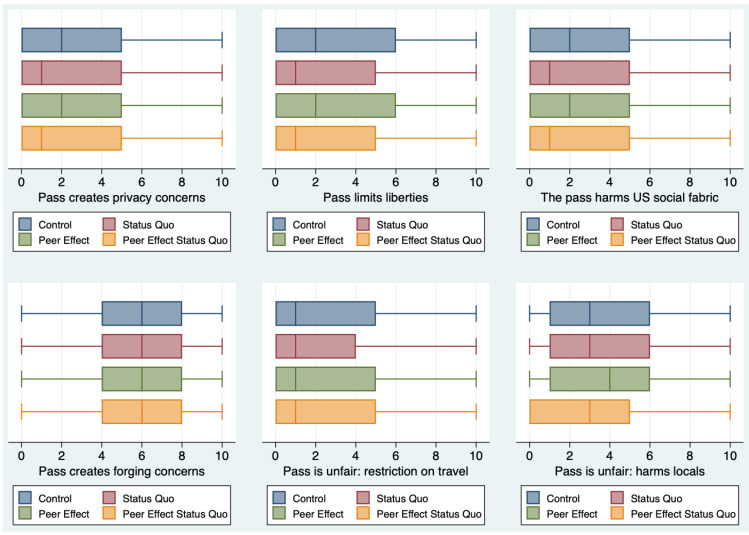
Summary of results for the statements meant to capture negative attributes of the COVID pass (statements 4–9). The boxplots show the distribution of responses in the Control, Status Quo, Peer Effect, and Peer Effect + Status Quo groups without any additional control. A lower value corresponds to more favorable attitudes towards the pass.

Overall, for virtually all statements, we find that the two treatments used together have a stronger impact. Figure 4 and Figure 5 summarize our findings.

To further ensure that our results are robust, we carried out a post hoc test to estimate the marginal effect of the combination of the two nudges relative to the effect of each one separately. The results are included in supplementary materials and are consistent with the results reported in Table 3.

### 4.4. Intention to get Vaccinated

Lastly, we turn to the investigation of whether the treatments affect the intention to get vaccinated if a COVID pass were introduced. The results are indicated in Table 4. We carried out the analysis considering the respondents who had completed a cycle of vaccination, the respondents who had received only one dose, and respondents who had not received a dose yet separately.

We find that the treatments do not have a negative impact on the intention to get vaccinated in case a COVID pass were introduced for any of these groups. Moreover, we find that the treatments do not have a negative impact also when aggregating the three groups (Table 4 and Tables S15–S18).

Thus, the introduction of a COVID pass is unlikely to affect intentions to get vaccinated negatively.

## 5. Discussion

Our experiment revealed that the status quo bias and peer effect can be used together as an effective means to increase support for the COVID pass without reducing intentions to get vaccinated. These findings have both immediate policy implications and broader theoretical implications.

### 5.1. Policy Implications

COVID passes could play a crucial role in restarting large-scale international travel. However, for them to be successful, they must be perceived as both important and fair by the general public. Our results can aid policymakers in ensuring that these conditions are met.

Flying without being vaccinated imposes significant externalities on the country of destination, the other passengers on the flight, and society at large. As noted by Sunstein [35], nudges aimed at mitigating a negative externality are not particularly controversial from an ethical perspective. The only relevant issue is whether they are effective. From this perspective, we find that both the status quo and peer effect are highly effective in increasing the support for the COVID pass. Most importantly, our results suggest that policymakers who want to increase the support for the COVID pass should rely on the two nudges simultaneously, as this allows them to both increase the perceived importance and the perceived fairness of the COVID pass. In fact, while the relationship between the nudges is not synergistic but weakly additive, the cost of each nudge is likely to be minimal and their combined effect is larger. However, policymakers have often neglected to state that requiring proof of vaccination for international travel is not unprecedented, and that in the case of COVID, it would be opposed by only a minority of people (e.g., [54]).

Nevertheless, in some contexts, policymakers and private companies might not have the possibility to employ both nudges. For instance, according to the Advertising Research Foundation (ARF), six-second TV advertising slots are very effective in commanding more attention per second than longer advertisements [55]. For such short communications, policymakers and private companies might have to focus on a single nudge. In this case, if they intend to emphasize the importance of the COVID pass, they should rely on the peer effect nudge, whereas if they intend to flag the fairness of the pass, they should build on the status quo bias.

### 5.2. Theoretical Implications

At a theoretical level, our work contributes to two strands of literature that are gaining momentum: (*i*) the study of interactions among nudges and (*ii*) behavioral spillovers triggered by nudges.

The literature on the interaction among nudges is in its infancy and, to the best of our knowledge, there are no studies testing the interaction between the peer effect and the status quo bias, especially in health-related domains. As these nudges are among the most widely studied in the literature and are effective in many domains, it is important to understand whether they can be used together or if their joint use is likely to backfire. Our results reveal that, at least in relation to COVID vaccine pass, the status quo and the peer effect nudges are effective together and their effects are weakly additive.

While our results refer to a very unique setting, there are reasons to believe that the status quo bias and the peer effect could often be in a weakly additive relationship. First, by definition, a peer effect nudge such as the one used in our experiment can be implemented only for policies that already have a fair amount of support. Therefore, it is likely that, for these policies, the marginal returns from policy pressure diminish at a fairly fast rate. This argument holds both at the society level, as greater support implies that there are only a few people left to convince, and at the individual level, where support is already high and cannot further increase by much. This might make it unlikely to observe a synergistic relationship when using the status quo in combination with other nudges.

Second, while they exploit different mechanisms, there is a connection between the two forms of nudges, as they both rely on social influence. Social psychology distinguishes between two main forms of social influence: informational social influence (telling people what is commonly done) and normative social influence (informing them about what is widely approved) [33,34]. The nudges we implemented in this paper are likely to stimulate both forms of social influence. In fact, the status quo bias suggests that, *in the past*, there was sufficient support for the policy that it was implemented, whereas the peer effect suggests that there is widespread support for the policy in question *today*. The interaction of informational and normative social influence implicitly pushes the individual in the same direction, making it reasonable to expect some compounding in the effects of the two nudges.

Moreover, as neither of the two interventions relies on monetary incentives, it is unlikely that extrinsic motivations would crowd out the intrinsic motivations triggered by the nudges. For these reasons, we suggest that combining the status quo and peer effect is highly unlikely to backfire.

In addition, we extend the literature on behavioral spillovers by investigating whether nudges on highly topical issues can generate a backlash on other key behaviors. We observe that, in this context, neither of the treatments had a negative impact on the intention to get vaccinated, thus suggesting that negative spillovers are not going to offset the positive impact of these nudges.

### 5.3. Limitations of the Analysis and Future Research

From a methodological standpoint, our study suffers from two limitations. First, we carried out an online experiment, and hence—like with every online experiment—one can question its external validity. This is partially alleviated by the heterogeneous composition of our sample and its large size. Second, while we rely on a large sample that closely matches the U.S. population along key variables, our sample is not representative. However, online experiments with non-representative samples are widely used across many disciplines and have been proven to be a reliable source of information, with a good degree of generalizability [56]. Moreover, the magnitude of the coefficients is large and robust to different sets of controls, suggesting that our results are likely to be informative even if the sample is not representative.

Additionally, the effect of our treatments is also likely to be context-dependent. For instance, it is possible that the impact of the peer effect treatment might change once the percentage of people opposing the COVID pass changes. Therefore, it is of key importance to implement these nudges before too many people start to oppose the COVID pass. Lastly, we do not include a manipulation check in our experiment.

## Figures and Tables

**Table 1:** List of statements with their respective number and summary statistics (mean and standard deviation). Participants were asked to state their agreement with the following sentences on a scale from 0 to 10.

St. Nr.	Statement	(Total)		(Control)		(Status Quo)		(Peer Effect)		(SQ+ PE)	
		Mean	SD	Mean	SD	Mean	SD	Mean	SD	Mean	SD
1	COVID pass is important to fight COVID-19	6.54	3.35	6.14	3.33	6.62	3.30	6.59	3.42	6.80	3.32
2	A COVID pass can help to prevent new variants of COVID-19 that might render current COVID-19 vaccines ineffective	6.29	3.40	5.90	3.39	6.22	3.40	6.34	3.41	6.68	3.37
3	A COVID pass is key to returning to normal quickly and safely	6.16	3.38	5.78	3.40	6.19	3.29	6.17	3.44	6.52	3.37
4	A COVID pass is an extreme limitation to the individual liberties of Americans	3.11	3.45	3.29	3.44	2.91	3.39	3.31	3.58	2.90	3.37
5	A COVID pass could harm the U.S. social fabric	2.91	3.32	3.11	3.34	2.83	3.29	3.04	3.36	2.64	3.27
6	A COVID pass poses severe dangers to Americans’ data privacy	3.03	3.36	3.21	3.31	2.88	3.35	3.17	3.42	2.86	3.34
7	It is unfair that people with a COVID pass can travel internationally, while individuals without it cannot	2.72	3.38	2.73	3.39	2.51	3.25	2.95	3.49	2.70	3.38
8	Allowing people with a COVID pass to travel to countries with lower access to vaccines and potentially come into contact with unvaccinated locals is unfair	3.66	3.21	3.68	3.17	3.55	3.14	3.91	3.27	3.49	3.25
9	A COVID pass could be easily forged	5.88	2.97	5.95	3.00	5.87	2.93	5.95	2.95	5.74	2.99
10	A COVID pass would induce more people to get vaccinated	6.17	2.81	6.10	2.76	6.19	2.79	6.11	2.92	6.29	2.75
11	Only people with a COVID pass should be allowed to board international flights	6.30	3.52	6.10	3.52	6.31	3.48	6.35	3.55	6.46	3.54
12	If a COVID pass were implemented, I would intentionally infect myself with COVID-19 to obtain it	0.57	1.64	0.58	1.68	0.59	1.67	0.55	1.58	0.56	1.63
13	Overall, the pros of requiring a COVID pass for international traveling outweigh the cons	6.65	3.33	6.38	3.36	6.79	3.22	6.52	3.40	6.89	3.32
Obs		3993		999		1000		999		995	

**Table 2:** Sample balance: the frequency table reports the number, percentage, and cumulative percentage of respondents for the following variables: political orientation, gender, income, education, age, employment, and race. Column 1 shows the distribution for the Control group, Column 2 shows the distribution for the Status Quo group, Column 3 shows the distribution for the Peer Effect group, Column 4 shows the distribution for the Status Quo + Peer Effect group (“PE + Status Quo”), and Column 5 shows the overall distribution in the sample.

	Group														
	Control			Status Quo			Peer Effect			PE + Status Quo			Total		
	No.	Col %	Cum %	No.	Col %	**Cum** %	No.	Col %	Cum %	No.	Col %	Cum %	No.	Col %	Cum %
**Political Orientation**															
Republican	167	16.7	16.7	165	16.5	16.5	163	16.3	16.3	163	16.4	16.4	658	16.5	16.5
Democrat	571	57.2	73.9	575	57.5	74.0	573	57.4	73.7	550	55.3	71.7	2269	56.8	73.3
Other or No Strong Preference	261	26.1	100.0	260	26.0	100.0	263	26.3	100.0	282	28.3	100.0	1066	26.7	100.0
**Total**	999	100.0		1000	100.0		999	100.0		995	100.0		3993	100.0	
**Gender**															
Other/Prefer not to declare	32	3.2	3.2	20	2.0	2.0	30	3.0	3.0	23	2.3	2.3	105	2.6	2.6
Female	551	55.2	58.4	582	58.2	60.2	607	60.8	63.8	553	55.6	57.9	2293	57.4	60.1
Male	416	41.6	100.0	398	39.8	100.0	362	36.2	100.0	419	42.1	100.0	1595	39.9	100.0
**Income**															
Less than $10,000	56	5.6	5.6	53	5.3	5.3	66	6.6	6.6	62	6.2	6.2	237	5.9	5.9
$10,000 to $19,999	63	6.3	11.9	64	6.4	11.7	62	6.2	12.8	87	8.8	15.0	276	6.9	12.9
$20,000 to $29,999	93	9.3	21.2	85	8.5	20.2	104	10.4	23.3	82	8.3	23.3	364	9.1	22.0
$30,000 to $39,999	81	8.1	29.4	99	9.9	30.1	95	9.5	32.8	96	9.7	32.9	371	9.3	31.3
$40,000 to $49,999	106	10.6	40.0	106	10.6	40.7	82	8.2	41.0	71	7.2	40.1	365	9.2	40.5
$50,000 to $59,999	112	11.2	51.2	111	11.1	51.9	93	9.3	50.4	82	8.3	48.3	398	10.0	50.4
$60,000 to $69,999	74	7.4	58.6	76	7.6	59.5	81	8.1	58.5	82	8.3	56.6	313	7.9	58.3
$70,000 to $79,999	85	8.5	67.1	66	6.6	66.1	75	7.5	66.0	90	9.1	65.7	316	7.9	66.2
$80,000 to $89,999	42	4.2	71.3	55	5.5	71.6	46	4.6	70.6	58	5.8	71.5	201	5.0	71.3
$80,000 to $89,999	54	5.4	76.8	57	5.7	77.3	55	5.5	76.1	53	5.3	76.8	219	5.5	76.7
$90,000 to $99,999	155	15.5	92.3	142	14.2	91.5	139	13.9	90.1	151	15.2	92.0	587	14.7	91.5
$150,000 or more	77	7.7	100.0	85	8.5	100.0	99	9.9	100.0	79	8.0	100.0	340	8.5	100.0
**Education**															
Less than high school degree	8	0.8	0.8	7	0.7	0.7	14	1.4	1.4	7	0.7	0.7	36	0.9	0.9
High school graduate (diploma or equivalent)	103	10.3	11.1	104	10.4	11.1	102	10.2	11.6	109	11.0	11.7	418	10.5	11.4
Some college but no degree	206	20.6	31.7	230	23.0	34.1	230	23.0	34.6	227	22.8	34.5	893	22.4	33.8
Associate degree in college (2-year)	93	9.3	41.0	84	8.4	42.5	109	10.9	45.5	111	11.2	45.7	397	9.9	43.7
Bachelor’s degree in college	400	40.0	81.1	373	37.3	79.9	347	34.7	80.3	371	37.3	83.0	1491	37.4	81.1
Master’s degree or Professional Degree (JD, MD)	176	17.6	98.7	177	17.7	97.6	169	16.9	97.2	148	14.9	97.9	670	16.8	97.8
Doctoral degree	13	1.3	100.0	24	2.4	100.0	28	2.8	100.0	21	2.1	100.0	86	2.2	100.0
**Age**															
18–25 years old	253	25.3	25.3	250	25.0	25.0	218	21.8	21.8	240	24.1	24.1	961	24.1	24.1
26–35 years old	343	34.3	59.7	354	35.4	60.4	358	35.8	57.7	317	31.9	56.0	1372	34.4	58.4
36–45 years old	185	18.5	78.2	179	17.9	78.3	189	18.9	76.6	194	19.5	75.5	747	18.7	77.1
46–55 years old	91	9.1	87.3	109	10.9	89.2	101	10.1	86.7	108	10.9	86.3	409	10.2	87.4
56–65 years old	64	6.4	93.7	50	5.0	94.2	61	6.1	92.8	73	7.3	93.7	248	6.2	93.6
66–75 years old	21	2.1	95.8	19	1.9	96.1	24	2.4	95.2	31	3.1	96.8	95	2.4	96.0
>75 years old	42	4.2	100.0	39	3.9	100.0	48	4.8	100.0	32	3.2	100.0	161	4.0	100.0
**In full or part time employment**	659	66.0	100.0	642	64.2	100.0	649	65.0	100.0	643	64.6	100.0	2593	64.9	100.0
**Student**	106	10.6	100.0	117	11.7	100.0	127	12.7	100.0	120	12.1	100.0	470	11.8	100.0
**White**	741	74.2	100.0	761	76.1	100.0	730	73.1	100.0	709	71.3	100.0	2941	73.7	100.0

**Table 3:** OLS beta coefficients deriving from regressions with the statements as the dependent variable and a binary variable to measure the impact of being in the treatment groups with respect to the Control group. The regressions are ran controlling for demographics, vaccination status, frequency of travel and trust levels of the participants. Tables of the regressions output are included in supplementary materials (Tables S2–S14).

Nr. Stmt	N	$\beta_{\mathrm{SQ}}$	$p_{\mathrm{SQ}}$	$\beta_{\mathrm{PE}}$	$p_{\mathrm{PE}}$	$\beta_{\mathrm{PE}+\mathrm{SQ}}$	$p_{\mathrm{PE}+\mathrm{SQ}}$
1	3705	0.525	<0.001	0.56	<0.001	0.707	<0.001
2	3704	0.404	0.002	0.544	<0.001	0.88	<0.001
3	3696	0.471	<0.001	0.48	<0.001	0.792	<0.001
4	3656	−0.413	0.001	−0.101	0.438	−0.485	<0.001
5	3639	−0.308	0.014	−0.195	0.128	−0.527	<0.001
6	3636	−0.364	0.004	−0.188	0.137	−0.443	<0.001
7	3634	−0.331	0.008	0.0755	0.566	−0.134	0.302
8	3664	−0.207	0.137	0.175	0.218	−0.264	0.059
9	3703	−0.09	0.488	−0.0582	0.66	−0.221	0.094
10	3701	0.107	0.362	0.0308	0.797	0.17	0.141
11	3693	0.305	0.024	0.302	0.027	0.361	0.008
12	3579	0.0314	0.681	−0.0094	0.897	0.0046	0.951
13	3690	0.494	<0.001	0.24	0.055	0.592	<0.001

**Table 4:** OLS beta coefficients deriving from regressions with the intention to get vaccinated as the dependent variable and a binary variable to measure the impact of being in the treatment groups with respect to the Control group. The regressions are ran controlling for demographics, vaccination status, frequency of travel and trust levels of the participants. Tables of the regressions output are included in supplementary materials (Tables S2–S14).

Status	N	$\beta_{\mathrm{SQ}}$	$p_{\mathrm{SQ}}$	$\beta_{\mathrm{PE}}$	$p_{\mathrm{PE}}$	$\beta_{\mathrm{PE}+\mathrm{SQ}}$	$p_{\mathrm{PE}+\mathrm{SQ}}$
Unvaccinated	1158	0.0124	0.897	−0.12	0.238	−0.01	0.919
One Dose	433	−0.275	0.059	−0.0807	0.501	0.103	0.31
Vaccinated	2121	−0.03	0.494	0.007	0.879	−0.026	0.572
All Sample	3716	−0.0535	0.279	−0.07	0.169	−0.033	0.505

## Data Availability

The data to replicate the analysis will be made publicly available. The anonymized responses of the survey participants will be made available by the author on request.

## Supplementary Materials

The following are available at https://www.mdpi.com/article/10.3390/ijerph18168800/s1.
