# Supplementary material for: COVID-19 Vaccine Passport and International Traveling: The Combined Effect of Two Nudges on Americans’ Support for the Pass
Source: Int J Environ Res Public Health. 2021 Aug 20;18(16):8800. doi: 10.3390/ijerph18168800 (PMC8394660; doi:10.3390/ijerph18168800)
Supplement: Supplementary file 1 [file ijerph-18-08800-s001.zip › ijerph-1260696-supplementary.pdf]

Supplementary Material

1. Factor analysis

Table S1: Factor analysis results using the first factor to consider all the statements about the pass

	(1)<br>Scores for factor 1	(2)<br>Scores for factor 1	(3)<br>Scores for factor 1	(4)<br>Scores for factor 1	(5)<br>Scores for factor 1	(6)<br>Scores for factor 1
Status Quo	0.119***	0.119***	0.137***	0.137***	0.141***	0.143***
	(0.008)	(0.008)	(0.001)	(0.001)	(0.000)	(0.000)
Peer Effect	0.0535	0.0535	0.0622	0.0622	0.100***	0.1000***
	(0.246)	(0.246)	(0.141)	(0.141)	(0.004)	(0.004)
Peer Effect Status Quo	0.141***	0.141***	0.160***	0.160***	0.180***	0.180***
	(0.002)	(0.002)	(0.000)	(0.000)	(0.000)	(0.000)
Age			0.00175	0.00175	0.0000631	0.000360
			(0.197)	(0.197)	(0.956)	(0.751)
Income			0.00885**	0.00885**	0.000206	-0.00134
			(0.045)	(0.045)	(0.957)	(0.729)
Female			-0.0115	-0.0115	0.0268	0.0300
			(0.702)	(0.702)	(0.285)	(0.233)
White			-0.0256	-0.0256	-0.0448	-0.0395
			(0.455)	(0.455)	(0.118)	(0.170)
Political Scale			-0.184***	-0.184***	-0.125***	-0.124***
			(0.000)	(0.000)	(0.000)	(0.000)
Education			0.0681***	0.0681***	0.0353***	0.0297***
			(0.000)	(0.000)	(0.001)	(0.005)
In full or part time employment			-0.0165	-0.0165	-0.0483	-0.0559*
			(0.677)	(0.677)	(0.137)	(0.088)
Student			0.236***	0.236***	0.100**	0.0932**
			(0.000)	(0.000)	(0.032)	(0.047)
Republican			-0.317***	-0.317***	-0.161***	-0.165***
			(0.000)	(0.000)	(0.001)	(0.000)
Trust in Federal Government					0.0391***	0.0388***
					(0.000)	(0.000)
Trust in State Government					-0.0226***	-0.0231***
					(0.001)	(0.001)
Trust in CDC					0.149***	0.149***
					(0.000)	(0.000)
Trust in Pharmaceutical companies					0.0258***	0.0257***
					(0.000)	(0.000)
Trust in Tech companies					0.00160	0.000701
					(0.835)	(0.927)
Frequency of travel						0.0277**
						(0.024)
Constant	-0.0781**	-0.0781**	0.0475	0.0475	-0.918***	-0.923***
	(0.013)	(0.013)	(0.550)	(0.550)	(0.000)	(0.000)
Observations	3639	3639	3489	3489	3461	3461
Adjusted R^2	0.002	0.002	0.220	0.220	0.467	0.467

p-values in parentheses
* $p < 0.10$, ** $p < 0.05$, *** $p < 0.01$

```
Factor analysis/correlation                      Number of obs    =      3,639
    Method: principal factors                    Retained factors =          5
    Rotation: (unrotated)                        Number of params =         55
```

Factor	Eigenvalue	Difference	Proportion	Cumulative
Factor1	7.18628	6.41494	0.9111	0.9111
Factor2	0.77134	0.56656	0.0978	1.0089
Factor3	0.20478	0.02751	0.0260	1.0349
Factor4	0.17727	0.07865	0.0225	1.0574
Factor5	0.09862	0.11482	0.0125	1.0699
Factor6	-0.01621	0.02471	-0.0021	1.0678
Factor7	-0.04092	0.01193	-0.0052	1.0626
Factor8	-0.05285	0.00414	-0.0067	1.0559
Factor9	-0.05699	0.00850	-0.0072	1.0487
Factor10	-0.06549	0.00325	-0.0083	1.0404
Factor11	-0.06875	0.03420	-0.0087	1.0317
Factor12	-0.10295	0.04400	-0.0131	1.0186
Factor13	-0.14695	.	-0.0186	1.0000

LR test: independent vs. saturated: chi2(78) = 4.0e+04 Prob>chi2 = 0.0000

Factor loadings (pattern matrix) and unique variances

Variable	Factor1	Factor2	Factor3	Factor4	Factor5	Uniqueness
pass_impor~e	0.8847	0.2785	0.0718	-0.0427	-0.1010	0.1227
pass_preve~d	0.8057	0.3012	0.0467	-0.0603	-0.1310	0.2372
pass_norma~y	0.8668	0.2857	0.0181	-0.0448	-0.0586	0.1613
pass_limit~y	-0.8783	0.2984	-0.1140	0.0523	-0.0269	0.1230
pass_socia~c	-0.8507	0.2912	-0.1094	0.1176	-0.0344	0.1644
pass_privacy	-0.8421	0.2603	-0.0801	0.1173	-0.0774	0.1970
pass_unfai~l	-0.8231	0.2474	0.0671	-0.1790	0.0762	0.2189
pass_unfai~s	-0.5092	0.2511	0.2729	-0.0142	0.0779	0.5969
pass_forge	-0.3603	0.0362	0.2641	0.1664	0.0005	0.7715
pass_induc~e	0.5484	0.2622	-0.1169	-0.0629	0.1427	0.5925
pass_fligh~r	0.8280	0.1359	-0.0182	0.1952	0.0927	0.2489
pass_inten~n	-0.0973	0.2293	-0.0527	-0.1087	0.0970	0.9139
pass_prosc~s	0.8582	0.1199	-0.0165	0.1603	0.0984	0.2135

2. Regression tables for the statements capturing respondents' support for the pass

Table S2: Determinants of agreement with the importance of a COVID pass to fight the pandemic

	(1) Pass importance	(2) Pass importance	(3) Pass importance	(4) Pass importance	(5) Pass importance	(6) Pass importance
Status Quo	0.478***	0.478***	0.507***	0.507***	0.519***	0.525***
	(0.001)	(0.001)	(0.000)	(0.000)	(0.000)	(0.000)
Peer Effect	0.449***	0.449***	0.489***	0.489***	0.564***	0.560***
	(0.003)	(0.003)	(0.001)	(0.001)	(0.000)	(0.000)
Peer Effect Status Quo	0.665***	0.665***	0.713***	0.713***	0.711***	0.707***
	(0.000)	(0.000)	(0.000)	(0.000)	(0.000)	(0.000)
Age			0.0100**	0.0100**	0.00385	0.00542
			(0.028)	(0.028)	(0.331)	(0.171)
Income			0.0321**	0.0321**	-0.00303	-0.0114
			(0.038)	(0.038)	(0.825)	(0.408)
Female			0.0321	0.0321	0.165*	0.182**
			(0.755)	(0.755)	(0.061)	(0.039)
White			-0.197*	-0.197*	-0.245**	-0.215**
			(0.097)	(0.097)	(0.017)	(0.036)
Political Scale			-0.482***	-0.482***	-0.297***	-0.294***
			(0.000)	(0.000)	(0.000)	(0.000)
Education			0.247***	0.247***	0.128***	0.0972***
			(0.000)	(0.000)	(0.000)	(0.010)
In full or part time employment			-0.00658	-0.00658	-0.110	-0.148
			(0.961)	(0.961)	(0.330)	(0.191)
Student			0.855***	0.855***	0.394**	0.360**
			(0.000)	(0.000)	(0.018)	(0.032)
Republican			-1.080***	-1.080***	-0.490***	-0.516***
			(0.000)	(0.000)	(0.002)	(0.001)
Trust in Federal Government					0.172***	0.170***
					(0.000)	(0.000)
Trust in State Government					-0.0713***	-0.0742***
					(0.003)	(0.002)
Trust in CDC					0.452***	0.455***
					(0.000)	(0.000)
Trust in Pharmaceutical companies					0.0521**	0.0517**
					(0.043)	(0.044)
Trust in Tech companies					0.0662**	0.0616**
					(0.012)	(0.019)
Frequency of travel						0.150***
						(0.000)
Constant	6.139***	6.139***	5.949***	5.949***	2.798***	2.770***
	(0.000)	(0.000)	(0.000)	(0.000)	(0.000)	(0.000)
Observations	3977	3977	3784	3784	3705	3705
Adjusted R^2	0.005	0.005	0.153	0.153	0.387	0.388

p-values in parentheses

* $p < 0.10$, ** $p < 0.05$, *** $p < 0.01$

Table S3: Determinants of agreement with the COVID pass helping to reduce the spread of new variants of the virus

	(1) Pass prevents the spread	(2) Pass prevents the spread	(3) Pass prevents the spread	(4) Pass prevents the spread	(5) Pass prevents the spread	(6) Pass prevents the spread
Status Quo	0.326**	0.326**	0.393***	0.393***	0.399***	0.404***
	(0.032)	(0.032)	(0.007)	(0.007)	(0.003)	(0.002)
Peer Effect	0.444***	0.444***	0.480***	0.480***	0.547***	0.544***
	(0.004)	(0.004)	(0.001)	(0.001)	(0.000)	(0.000)
Peer Effect Status Quo	0.787***	0.787***	0.874***	0.874***	0.882***	0.880***
	(0.000)	(0.000)	(0.000)	(0.000)	(0.000)	(0.000)
Age			0.00527	0.00527	0.0000639	0.00115
			(0.259)	(0.259)	(0.988)	(0.786)
Income			0.0351**	0.0351**	0.00649	0.000665
			(0.026)	(0.026)	(0.656)	(0.964)
Female			-0.109	-0.109	0.0143	0.0259
			(0.301)	(0.301)	(0.880)	(0.784)
White			-0.0234	-0.0234	-0.0766	-0.0562
			(0.847)	(0.847)	(0.486)	(0.611)
Political Scale			-0.487***	-0.487***	-0.316***	-0.314***
			(0.000)	(0.000)	(0.000)	(0.000)
Education			0.196***	0.196***	0.0863**	0.0652
			(0.000)	(0.000)	(0.025)	(0.100)
In full or part time employment			-0.125	-0.125	-0.243**	-0.269**
			(0.353)	(0.353)	(0.042)	(0.025)
Student			0.663***	0.663***	0.240	0.216
			(0.001)	(0.001)	(0.188)	(0.238)
Republican			-0.968***	-0.968***	-0.454***	-0.472***
			(0.000)	(0.000)	(0.007)	(0.005)
Trust in Federal Government					0.162***	0.162***
					(0.000)	(0.000)
Trust in State Government					-0.0692***	-0.0712***
					(0.008)	(0.007)
Trust in CDC					0.407***	0.409***
					(0.000)	(0.000)
Trust in Pharmaceutical companies					0.0501*	0.0500*
					(0.072)	(0.073)
Trust in Tech companies					0.0413	0.0380
					(0.132)	(0.166)
Frequency of travel						0.104**
						(0.022)
Constant	5.897***	5.897***	6.104***	6.104***	3.306***	3.286***
	(0.000)	(0.000)	(0.000)	(0.000)	(0.000)	(0.000)
Observations	3974	3974	3781	3781	3704	3704
Adjusted R^2	0.006	0.006	0.141	0.141	0.320	0.321

p-values in parentheses

* $p < 0.10$, ** $p < 0.05$, *** $p < 0.01$

Table S4: Determinants of agreement with the COVID pass helping the return to normalcy

	(1)<br>Pass helps return to normalcy	(2)<br>Pass helps return to normalcy	(3)<br>Pass helps return to normalcy	(4)<br>Pass helps return to normalcy	(5)<br>Pass helps return to normalcy	(6)<br>Pass helps return to normalcy
Status Quo	0.409***	0.409***	0.470***	0.470***	0.462***	0.471***
	(0.006)	(0.006)	(0.001)	(0.001)	(0.000)	(0.000)
Peer Effect	0.395**	0.395**	0.447***	0.447***	0.487***	0.480***
	(0.010)	(0.010)	(0.003)	(0.003)	(0.000)	(0.000)
Peer Effect Status Quo	0.737***	0.737***	0.812***	0.812***	0.799***	0.792***
	(0.000)	(0.000)	(0.000)	(0.000)	(0.000)	(0.000)
Age			0.00541	0.00541	-0.00129	0.00111
			(0.247)	(0.247)	(0.751)	(0.785)
Income			0.0383**	0.0383**	0.00239	-0.0105
			(0.015)	(0.015)	(0.864)	(0.457)
Female			-0.000601	-0.000601	0.139	0.164*
			(0.995)	(0.995)	(0.127)	(0.071)
White			-0.149	-0.149	-0.155	-0.110
			(0.215)	(0.215)	(0.140)	(0.293)
Political Scale			-0.475***	-0.475***	-0.302***	-0.297***
			(0.000)	(0.000)	(0.000)	(0.000)
Education			0.198***	0.198***	0.0885**	0.0416
			(0.000)	(0.000)	(0.017)	(0.275)
In full or part time employment			0.0149	0.0149	-0.100	-0.160
			(0.913)	(0.913)	(0.393)	(0.173)
Student			0.687***	0.687***	0.251	0.195
			(0.000)	(0.000)	(0.155)	(0.269)
Republican			-0.960***	-0.960***	-0.437***	-0.478***
			(0.000)	(0.000)	(0.006)	(0.002)
Trust in Federal Government					0.152***	0.150***
					(0.000)	(0.000)
Trust in State Government					-0.0726***	-0.0771***
					(0.004)	(0.002)
Trust in CDC					0.428***	0.432***
					(0.000)	(0.000)
Trust in Pharmaceutical companies					0.109***	0.108***
					(0.000)	(0.000)
Trust in Tech companies					0.0672**	0.0603**
					(0.013)	(0.026)
Frequency of travel						0.231***
						(0.000)
Constant	5.779***	5.779***	5.857***	5.857***	2.747***	2.707***
	(0.000)	(0.000)	(0.000)	(0.000)	(0.000)	(0.000)
Observations	3965	3965	3772	3772	3696	3696
Adjusted R^2	0.005	0.005	0.137	0.137	0.367	0.372

p-values in parentheses

* $p < 0.10$, ** $p < 0.05$, *** $p < 0.01$

Table S5: Determinants of agreement with the COVID pass being a limitation of liberties

	(1)<br>Pass limits liberties	(2)<br>Pass limits liberties	(3)<br>Pass limits liberties	(4)<br>Pass limits liberties	(5)<br>Pass limits liberties	(6)<br>Pass limits liberties
Status Quo	-0.372**	-0.372**	-0.388***	-0.388***	-0.409***	-0.413***
	(0.016)	(0.016)	(0.005)	(0.005)	(0.001)	(0.001)
Peer Effect	0.0294	0.0294	-0.0141	-0.0141	-0.103	-0.101
	(0.853)	(0.853)	(0.922)	(0.922)	(0.433)	(0.438)
Peer Effect Status Quo	-0.383**	-0.383**	-0.452***	-0.452***	-0.486***	-0.485***
	(0.013)	(0.013)	(0.001)	(0.001)	(0.000)	(0.000)
Age			0.00395	0.00395	0.00844**	0.00771*
			(0.388)	(0.388)	(0.039)	(0.059)
Income			-0.0249*	-0.0249*	0.000415	0.00421
			(0.097)	(0.097)	(0.976)	(0.767)
Female			0.0332	0.0332	-0.0814	-0.0891
			(0.742)	(0.742)	(0.369)	(0.326)
White			0.102	0.102	0.160	0.147
			(0.385)	(0.385)	(0.134)	(0.173)
Political Scale			0.647***	0.647***	0.460***	0.459***
			(0.000)	(0.000)	(0.000)	(0.000)
Education			-0.276***	-0.276***	-0.175***	-0.161***
			(0.000)	(0.000)	(0.000)	(0.000)
In full or part time employment			0.175	0.175	0.257**	0.275**
			(0.188)	(0.188)	(0.029)	(0.020)
Student			-0.816***	-0.816***	-0.396**	-0.379**
			(0.000)	(0.000)	(0.017)	(0.022)
Republican			1.040***	1.040***	0.587***	0.599***
			(0.000)	(0.000)	(0.001)	(0.001)
Trust in Federal Government					-0.0827***	-0.0822***
					(0.006)	(0.006)
Trust in State Government					0.0692***	0.0703***
					(0.005)	(0.005)
Trust in CDC					-0.462***	-0.464***
					(0.000)	(0.000)
Trust in Pharmaceutical companies					-0.0686**	-0.0683**
					(0.011)	(0.011)
Trust in Tech companies					0.0296	0.0318
					(0.284)	(0.251)
Frequency of travel						-0.0689
						(0.121)
Constant	3.286***	3.286***	2.546***	2.546***	5.245***	5.259***
	(0.000)	(0.000)	(0.000)	(0.000)	(0.000)	(0.000)
Observations	3908	3908	3727	3727	3656	3656
Adjusted R^2	0.002	0.002	0.225	0.225	0.383	0.383

p-values in parentheses

* $p < 0.10$, ** $p < 0.05$, *** $p < 0.01$

Table S6: Determinants of agreement with the COVID pass harming the US social fabric

	(1)<br>The pass harms US social fabric	(2)<br>The pass harms US social fabric	(3)<br>The pass harms US social fabric	(4)<br>The pass harms US social fabric	(5)<br>The pass harms US social fabric	(6)<br>The pass harms US social fabric
Status Quo	-0.280*	-0.280*	-0.293**	-0.293**	-0.305**	-0.308**
	(0.063)	(0.063)	(0.036)	(0.036)	(0.015)	(0.014)
Peer Effect	-0.0689	-0.0689	-0.104	-0.104	-0.195	-0.195
	(0.650)	(0.650)	(0.460)	(0.460)	(0.127)	(0.128)
Peer Effect Status Quo	-0.468***	-0.468***	-0.498***	-0.498***	-0.528***	-0.527***
	(0.002)	(0.002)	(0.000)	(0.000)	(0.000)	(0.000)
Age			-0.00272	-0.00272	0.00162	0.00109
			(0.553)	(0.553)	(0.694)	(0.792)
Income			-0.0132	-0.0132	0.0132	0.0160
			(0.372)	(0.372)	(0.334)	(0.251)
Female			-0.0776	-0.0776	-0.184**	-0.190**
			(0.438)	(0.438)	(0.040)	(0.035)
White			0.257**	0.257**	0.310***	0.300***
			(0.024)	(0.024)	(0.003)	(0.004)
Political Scale			0.566***	0.566***	0.393***	0.392***
			(0.000)	(0.000)	(0.000)	(0.000)
Education			-0.217***	-0.217***	-0.121***	-0.111***
			(0.000)	(0.000)	(0.001)	(0.004)
In full or part time employment			0.129	0.129	0.201*	0.214*
			(0.325)	(0.325)	(0.086)	(0.070)
Student			-0.824***	-0.824***	-0.425**	-0.413**
			(0.000)	(0.000)	(0.011)	(0.014)
Republican			0.975***	0.975***	0.514***	0.523***
			(0.000)	(0.000)	(0.002)	(0.002)
Trust in Federal Government					-0.0899***	-0.0894***
					(0.003)	(0.003)
Trust in State Government					0.0803***	0.0811***
					(0.001)	(0.001)
Trust in CDC					-0.431***	-0.432***
					(0.000)	(0.000)
Trust in Pharmaceutical companies					-0.0827***	-0.0825***
					(0.002)	(0.002)
Trust in Tech companies					0.0280	0.0295
					(0.319)	(0.294)
Frequency of travel						-0.0497
						(0.272)
Constant	3.112***	3.112***	2.462***	2.462***	4.998***	5.008***
	(0.000)	(0.000)	(0.000)	(0.000)	(0.000)	(0.000)
Observations	3878	3878	3707	3707	3639	3639
Adjusted R^2	0.002	0.002	0.190	0.190	0.346	0.346

p-values in parentheses

* $p < 0.10$, ** $p < 0.05$, *** $p < 0.01$

Table S7: Determinants of agreement with the COVID pass creating privacy concerns

	(1)<br>Pass creates<br>privacy concerns	(2)<br>Pass creates<br>privacy concerns	(3)<br>Pass creates<br>privacy concerns	(4)<br>Pass creates<br>privacy concerns	(5)<br>Pass creates<br>privacy concerns	(6)<br>Pass creates<br>privacy concerns
Status Quo	-0.324**	-0.324**	-0.357**	-0.357**	-0.363***	-0.364***
	(0.032)	(0.032)	(0.011)	(0.011)	(0.004)	(0.004)
Peer Effect	-0.0403	-0.0403	-0.0919	-0.0919	-0.188	-0.188
	(0.791)	(0.791)	(0.515)	(0.515)	(0.136)	(0.137)
Peer Effect Status Quo	-0.348**	-0.348**	-0.422***	-0.422***	-0.443***	-0.443***
	(0.021)	(0.021)	(0.003)	(0.003)	(0.000)	(0.000)
Age			0.00596	0.00596	0.0113***	0.0110***
			(0.195)	(0.195)	(0.007)	(0.008)
Income			-0.0291*	-0.0291*	-0.00143	0.0000602
			(0.051)	(0.051)	(0.918)	(0.997)
Female			-0.133	-0.133	-0.260***	-0.263***
			(0.190)	(0.190)	(0.004)	(0.004)
White			0.182	0.182	0.234**	0.228**
			(0.113)	(0.113)	(0.024)	(0.028)
Political Scale			0.591***	0.591***	0.416***	0.415***
			(0.000)	(0.000)	(0.000)	(0.000)
Education			-0.197***	-0.197***	-0.0944**	-0.0890**
			(0.000)	(0.000)	(0.012)	(0.021)
In full or part time employment			0.191	0.191	0.268**	0.275**
			(0.139)	(0.139)	(0.020)	(0.018)
Student			-0.549***	-0.549***	-0.149	-0.143
			(0.002)	(0.002)	(0.379)	(0.401)
Republican			0.933***	0.933***	0.475***	0.480***
			(0.000)	(0.000)	(0.006)	(0.005)
Trust in Federal Government					-0.0838***	-0.0836***
					(0.006)	(0.007)
Trust in State Government					0.0677***	0.0682***
					(0.007)	(0.007)
Trust in CDC					-0.431***	-0.432***
					(0.000)	(0.000)
Trust in Pharmaceutical companies					-0.0925***	-0.0924***
					(0.001)	(0.001)
Trust in Tech companies					0.00284	0.00372
					(0.920)	(0.895)
Frequency of travel						-0.0270
						(0.544)
Constant	3.208***	3.208***	2.249***	2.249***	4.885***	4.891***
	(0.000)	(0.000)	(0.000)	(0.000)	(0.000)	(0.000)
Observations	3882	3882	3707	3707	3636	3636
Adjusted R^2	0.001	0.001	0.195	0.195	0.361	0.360

p-values in parentheses
* $p < 0.10$, ** $p < 0.05$, *** $p < 0.01$

Table S8: Determinants of agreement with the COVID pass being unfair as it allows only people with a COVID pass to travel

	(1)<br>Pass is unfair:<br>travel restriction	(2)<br>Pass is unfair:<br>travel restriction	(3)<br>Pass is unfair:<br>travel restriction	(4)<br>Pass is unfair:<br>travel restriction	(5)<br>Pass is unfair:<br>travel restriction	(6)<br>Pass is unfair:<br>travel restriction
Status Quo	-0.223	-0.223	-0.310**	-0.310**	-0.333***	-0.331***
	(0.138)	(0.138)	(0.025)	(0.025)	(0.008)	(0.008)
Peer Effect	0.215	0.215	0.156	0.156	0.0761	0.0755
	(0.168)	(0.168)	(0.276)	(0.276)	(0.563)	(0.566)
Peer Effect Status Quo	-0.0301	-0.0301	-0.105	-0.105	-0.133	-0.134
	(0.845)	(0.845)	(0.461)	(0.461)	(0.305)	(0.302)
Age			-0.00664	-0.00664	-0.00344	-0.00287
			(0.137)	(0.137)	(0.399)	(0.484)
Income			-0.0440***	-0.0440***	-0.0238*	-0.0267*
			(0.003)	(0.003)	(0.093)	(0.061)
Female			0.0600	0.0600	-0.0275	-0.0215
			(0.552)	(0.552)	(0.765)	(0.816)
White			-0.117	-0.117	-0.0115	-0.00125
			(0.322)	(0.322)	(0.916)	(0.991)
Political Scale			0.619***	0.619***	0.439***	0.440***
			(0.000)	(0.000)	(0.000)	(0.000)
Education			-0.149***	-0.149***	-0.0551	-0.0659*
			(0.000)	(0.000)	(0.149)	(0.091)
In full or part time employment			0.108	0.108	0.158	0.144
			(0.408)	(0.408)	(0.182)	(0.229)
Student			-0.735***	-0.735***	-0.372**	-0.385**
			(0.000)	(0.000)	(0.029)	(0.024)
Republican			0.997***	0.997***	0.545***	0.536***
			(0.000)	(0.000)	(0.002)	(0.003)
Trust in Federal Government					-0.0575*	-0.0580*
					(0.056)	(0.054)
Trust in State Government					0.0672***	0.0663***
					(0.007)	(0.008)
Trust in CDC					-0.447***	-0.446***
					(0.000)	(0.000)
Trust in Pharmaceutical companies					-0.0405	-0.0408
					(0.135)	(0.132)
Trust in Tech companies					0.0379	0.0362
					(0.177)	(0.196)
Frequency of travel						0.0538
						(0.233)
Constant	2.734***	2.734***	2.196***	2.196***	4.629***	4.618***
	(0.000)	(0.000)	(0.000)	(0.000)	(0.000)	(0.000)
Observations	3872	3872	3697	3697	3634	3634
Adjusted R^2	0.001	0.001	0.199	0.199	0.333	0.333

p-values in parentheses
* $p < 0.10$, ** $p < 0.05$, *** $p < 0.01$

Table S9: Determinants of agreement with the COVID pass being unfair as it poses threats to locals when people with the COVID pass travel

	(1)	(2)	(3)	(4)	(5)	(6)
	Pass is unfair: harms locals	Pass is unfair: harms locals	Pass is unfair: harms locals	Pass is unfair: harms locals	Pass is unfair: harms locals	Pass is unfair: harms locals
Status Quo	-0.137	-0.137	-0.191	-0.191	-0.209	-0.207
	(0.336)	(0.336)	(0.181)	(0.181)	(0.132)	(0.137)
Peer Effect	0.225	0.225	0.190	0.190	0.176	0.175
	(0.121)	(0.121)	(0.189)	(0.189)	(0.215)	(0.218)
Peer Effect Status Quo	-0.192	-0.192	-0.247*	-0.247*	-0.263*	-0.264*
	(0.185)	(0.185)	(0.086)	(0.086)	(0.060)	(0.059)
Age			-0.0191***	-0.0191***	-0.0186***	-0.0180***
			(0.000)	(0.000)	(0.000)	(0.000)
Income			-0.0270*	-0.0270*	-0.0165	-0.0195
			(0.090)	(0.090)	(0.298)	(0.220)
Female			0.121	0.121	0.0635	0.0696
			(0.242)	(0.242)	(0.529)	(0.492)
White			-0.284**	-0.284**	-0.215*	-0.205*
			(0.019)	(0.019)	(0.072)	(0.087)
Political Scale			0.264***	0.264***	0.173***	0.174***
			(0.000)	(0.000)	(0.000)	(0.000)
Education			-0.0504	-0.0504	-0.00632	-0.0176
			(0.242)	(0.242)	(0.883)	(0.693)
In full or part time employment			-0.118	-0.118	-0.0896	-0.104
			(0.386)	(0.386)	(0.502)	(0.439)
Student			-0.580***	-0.580***	-0.353*	-0.366*
			(0.004)	(0.004)	(0.079)	(0.070)
Republican			0.788***	0.788***	0.498***	0.488***
			(0.000)	(0.000)	(0.006)	(0.007)
Trust in Federal Government					0.0133	0.0127
					(0.708)	(0.719)
Trust in State Government					0.0427	0.0417
					(0.152)	(0.162)
Trust in CDC					-0.283***	-0.282***
					(0.000)	(0.000)
Trust in Pharmaceutical companies					-0.0734**	-0.0735**
					(0.013)	(0.013)
Trust in Tech companies					0.0402	0.0384
					(0.186)	(0.206)
Frequency of travel						0.0552
						(0.261)
Constant	3.684***	3.684***	4.233***	4.233***	5.749***	5.739***
	(0.000)	(0.000)	(0.000)	(0.000)	(0.000)	(0.000)
Observations	3912	3912	3732	3732	3664	3664
Adjusted R^2	0.002	0.002	0.053	0.053	0.108	0.108

p-values in parentheses
* $p < 0.10$, ** $p < 0.05$, *** $p < 0.01$

Table S10: Determinants of agreement with forging a COVID pass being easy

	(1) Forging concerns	(2) Forging concerns	(3) Forging concerns	(4) Forging concerns	(5) Forging concerns	(6) Forging concerns
Status Quo	-0.0786	-0.0786	-0.0837	-0.0837	-0.0830	-0.0900
	(0.554)	(0.554)	(0.526)	(0.526)	(0.523)	(0.488)
Peer Effect	0.00288	0.00288	-0.0669	-0.0669	-0.0613	-0.0582
	(0.983)	(0.983)	(0.619)	(0.619)	(0.643)	(0.660)
Peer Effect Status Quo	-0.214	-0.214	-0.247*	-0.247*	-0.225*	-0.221*
	(0.112)	(0.112)	(0.066)	(0.066)	(0.089)	(0.094)
Age			0.0190***	0.0190***	0.0244***	0.0227***
			(0.000)	(0.000)	(0.000)	(0.000)
Income			0.0166	0.0166	0.0314**	0.0400***
			(0.258)	(0.258)	(0.033)	(0.007)
Female			0.477***	0.477***	0.415***	0.397***
			(0.000)	(0.000)	(0.000)	(0.000)
White			-0.127	-0.127	-0.172	-0.202*
			(0.261)	(0.261)	(0.124)	(0.071)
Political Scale			0.252***	0.252***	0.223***	0.220***
			(0.000)	(0.000)	(0.000)	(0.000)
Education			-0.143***	-0.143***	-0.0981**	-0.0670*
			(0.000)	(0.000)	(0.011)	(0.092)
In full or part time employment			-0.141	-0.141	-0.0949	-0.0565
			(0.249)	(0.249)	(0.435)	(0.644)
Student			-0.0336	-0.0336	0.147	0.183
			(0.854)	(0.854)	(0.422)	(0.321)
Republican			0.0119	0.0119	-0.0407	-0.0145
			(0.940)	(0.940)	(0.793)	(0.925)
Trust in Federal Government					-0.0829**	-0.0816**
					(0.014)	(0.016)
Trust in State Government					-0.0259	-0.0230
					(0.355)	(0.411)
Trust in CDC					-0.0408	-0.0437*
					(0.102)	(0.079)
Trust in Pharmaceutical companies					-0.116***	-0.116***
					(0.000)	(0.000)
Trust in Tech companies					0.0101	0.0150
					(0.716)	(0.591)
Frequency of travel						-0.153***
						(0.001)
Constant	5.952***	5.952***	5.082***	5.082***	5.755***	5.784***
	(0.000)	(0.000)	(0.000)	(0.000)	(0.000)	(0.000)
Observations	3972	3972	3784	3784	3703	3703
Adjusted R^2	0.000	0.000	0.046	0.046	0.084	0.086

p-values in parentheses

* $p < 0.10$, ** $p < 0.05$, *** $p < 0.01$

Table S11: Determinants of agreement with the COVID inducing more vaccinations

	(1) Pass induces vaccinations	(2) Pass induces vaccinations	(3) Pass induces vaccinations	(4) Pass induces vaccinations	(5) Pass induces vaccinations	(6) Pass induces vaccinations
Status Quo	0.0906	0.0906	0.106	0.106	0.103	0.107
	(0.467)	(0.467)	(0.398)	(0.398)	(0.384)	(0.362)
Peer Effect	0.0172	0.0172	0.0205	0.0205	0.0332	0.0308
	(0.893)	(0.893)	(0.873)	(0.873)	(0.782)	(0.797)
Peer Effect Status Quo	0.194	0.194	0.222*	0.222*	0.174	0.170
	(0.118)	(0.118)	(0.073)	(0.073)	(0.135)	(0.141)
Age			-0.0101**	-0.0101**	-0.0144***	-0.0131***
			(0.012)	(0.012)	(0.000)	(0.000)
Income			0.00840	0.00840	-0.0156	-0.0224*
			(0.541)	(0.541)	(0.232)	(0.087)
Female			-0.199**	-0.199**	-0.105	-0.0913
			(0.029)	(0.029)	(0.216)	(0.284)
White			-0.326***	-0.326***	-0.336***	-0.312***
			(0.002)	(0.002)	(0.001)	(0.002)
Political Scale			-0.189***	-0.189***	-0.0875***	-0.0849***
			(0.000)	(0.000)	(0.001)	(0.002)
Education			0.0826**	0.0826**	0.00511	-0.0194
			(0.027)	(0.027)	(0.885)	(0.593)
In full or part time employment			0.112	0.112	0.00918	-0.0213
			(0.345)	(0.345)	(0.934)	(0.849)
Student			0.559***	0.559***	0.240	0.211
			(0.001)	(0.001)	(0.152)	(0.208)
Republican			-0.355**	-0.355**	0.00467	-0.0169
			(0.030)	(0.030)	(0.974)	(0.907)
Trust in Federal Government					0.0735**	0.0726**
					(0.018)	(0.019)
Trust in State Government					0.00468	0.00235
					(0.858)	(0.928)
Trust in CDC					0.253***	0.255***
					(0.000)	(0.000)
Trust in Pharmaceutical companies					0.0397	0.0395
					(0.133)	(0.136)
Trust in Tech companies					0.0840***	0.0802***
					(0.002)	(0.003)
Frequency of travel						0.121***
						(0.003)
Constant	6.098***	6.098***	6.801***	6.801***	4.858***	4.837***
	(0.000)	(0.000)	(0.000)	(0.000)	(0.000)	(0.000)
Observations	3967	3967	3779	3779	3701	3701
Adjusted R^2	-0.000	-0.000	0.044	0.044	0.177	0.179

p-values in parentheses

* $p < 0.10$, ** $p < 0.05$, *** $p < 0.01$

Table S12: Determinants of agreement with requiring a COVID pass for international flights

	(1)<br>Pass required on international flights	(2)<br>Pass required on international flights	(3)<br>Pass required on international flights	(4)<br>Pass required on international flights	(5)<br>Pass required on international flights	(6)<br>Pass required on international flights
Status Quo	0.206	0.206	0.281*	0.281*	0.302**	0.305**
	(0.190)	(0.190)	(0.062)	(0.062)	(0.025)	(0.024)
Peer Effect	0.251	0.251	0.249	0.249	0.303**	0.302**
	(0.115)	(0.115)	(0.105)	(0.105)	(0.027)	(0.027)
Peer Effect Status Quo	0.357**	0.357**	0.386**	0.386**	0.363***	0.361***
	(0.025)	(0.025)	(0.011)	(0.011)	(0.008)	(0.008)
Age			0.0144***	0.0144***	0.0105**	0.0112***
			(0.003)	(0.003)	(0.014)	(0.009)
Income			0.0301*	0.0301*	-0.00224	-0.00585
			(0.072)	(0.072)	(0.883)	(0.703)
Female			-0.0625	-0.0625	0.0570	0.0644
			(0.566)	(0.566)	(0.557)	(0.507)
White			-0.185	-0.185	-0.212*	-0.200*
			(0.145)	(0.145)	(0.063)	(0.082)
Political Scale			-0.561***	-0.561***	-0.376***	-0.374***
			(0.000)	(0.000)	(0.000)	(0.000)
Education			0.127***	0.127***	0.0214	0.00835
			(0.004)	(0.004)	(0.595)	(0.840)
In full or part time employment			-0.0419	-0.0419	-0.124	-0.140
			(0.769)	(0.769)	(0.323)	(0.268)
Student			0.471**	0.471**	0.0659	0.0507
			(0.022)	(0.022)	(0.729)	(0.791)
Republican			-0.758***	-0.758***	-0.249	-0.260
			(0.000)	(0.000)	(0.137)	(0.120)
Trust in Federal Government					0.127***	0.126***
					(0.000)	(0.000)
Trust in State Government					-0.0678**	-0.0691**
					(0.012)	(0.011)
Trust in CDC					0.467***	0.469***
					(0.000)	(0.000)
Trust in Pharmaceutical companies					0.0796***	0.0794***
					(0.006)	(0.006)
Trust in Tech companies					0.00634	0.00427
					(0.833)	(0.887)
Frequency of travel						0.0642
						(0.179)
Constant	6.101***	6.101***	6.556***	6.556***	3.451***	3.439***
	(0.000)	(0.000)	(0.000)	(0.000)	(0.000)	(0.000)
Observations	3951	3951	3763	3763	3693	3693
Adjusted R^2	0.001	0.001	0.134	0.134	0.325	0.326

p-values in parentheses
* $p < 0.10$, ** $p < 0.05$, *** $p < 0.01$

Table S13: Determinants of agreement with intentionally getting infected with COVID to obtain the COVID pass

	(1)<br>Pass induces<br>intentional infections	(2)<br>Pass induces<br>intentional infections	(3)<br>Pass induces<br>intentional infections	(4)<br>Pass induces<br>intentional infections	(5)<br>Pass induces<br>intentional infections	(6)<br>Pass induces<br>intentional infections
Status Quo	0.0169	0.0169	0.0341	0.0341	0.0234	0.0314
	(0.826)	(0.826)	(0.656)	(0.656)	(0.760)	(0.681)
Peer Effect	-0.0284	-0.0284	-0.00272	-0.00272	-0.00731	-0.00942
	(0.705)	(0.705)	(0.971)	(0.971)	(0.921)	(0.897)
Peer Effect Status Quo	-0.0197	-0.0197	0.0167	0.0167	0.00688	0.00459
	(0.796)	(0.796)	(0.826)	(0.826)	(0.928)	(0.951)
Age			-0.0109***	-0.0109***	-0.0120***	-0.0102***
			(0.000)	(0.000)	(0.000)	(0.000)
Income			-0.0145*	-0.0145*	-0.0191**	-0.0281***
			(0.077)	(0.077)	(0.022)	(0.001)
Female			-0.118**	-0.118**	-0.114**	-0.0957*
			(0.031)	(0.031)	(0.036)	(0.077)
White			-0.0790	-0.0790	-0.0539	-0.0229
			(0.225)	(0.225)	(0.416)	(0.730)
Political Scale			0.0887***	0.0887***	0.0732***	0.0758***
			(0.000)	(0.000)	(0.000)	(0.000)
Education			0.0645***	0.0645***	0.0546**	0.0220
			(0.005)	(0.005)	(0.018)	(0.349)
In full or part time employment			0.236***	0.236***	0.233***	0.191***
			(0.000)	(0.000)	(0.000)	(0.001)
Student			0.0576	0.0576	0.0708	0.0332
			(0.514)	(0.514)	(0.432)	(0.714)
Republican			0.0366	0.0366	0.0392	0.0133
			(0.740)	(0.740)	(0.725)	(0.904)
Trust in Federal Government					0.0305	0.0294
					(0.116)	(0.129)
Trust in State Government					0.00308	0.0000730
					(0.810)	(0.995)
Trust in CDC					-0.0483***	-0.0455***
					(0.001)	(0.001)
Trust in Pharmaceutical companies					0.0271*	0.0266*
					(0.087)	(0.090)
Trust in Tech companies					0.0461***	0.0410***
					(0.002)	(0.005)
Frequency of travel						0.161***
						(0.000)
Constant	0.577***	0.577***	0.466***	0.466***	0.477***	0.445***
	(0.000)	(0.000)	(0.001)	(0.001)	(0.002)	(0.003)
Observations	3784	3784	3624	3624	3579	3579
Adjusted R^2	-0.001	-0.001	0.025	0.025	0.037	0.047

p-values in parentheses

* $p < 0.10$, ** $p < 0.05$, *** $p < 0.01$

Table S14: Determinants of agreement with the COVID pass having greater pros than cons

	(1)<br>Pros outweigh cons	(2)<br>Pros outweigh cons	(3)<br>Pros outweigh cons	(4)<br>Pros outweigh cons	(5)<br>Pros outweigh cons	(6)<br>Pros outweigh cons
Status Quo	0.412***	0.412***	0.459***	0.459***	0.491***	0.494***
	(0.005)	(0.005)	(0.001)	(0.001)	(0.000)	(0.000)
Peer Effect	0.146	0.146	0.154	0.154	0.242*	0.240*
	(0.336)	(0.336)	(0.285)	(0.285)	(0.054)	(0.055)
Peer Effect Status Quo	0.514***	0.514***	0.587***	0.587***	0.593***	0.592***
	(0.001)	(0.001)	(0.000)	(0.000)	(0.000)	(0.000)
Age			0.0155***	0.0155***	0.0120***	0.0127***
			(0.001)	(0.001)	(0.002)	(0.001)
Income			0.0520***	0.0520***	0.0242*	0.0205
			(0.001)	(0.001)	(0.077)	(0.135)
Female			-0.138	-0.138	-0.0407	-0.0332
			(0.170)	(0.170)	(0.644)	(0.707)
White			0.0761	0.0761	-0.0180	-0.00516
			(0.515)	(0.515)	(0.863)	(0.961)
Political Scale			-0.551***	-0.551***	-0.362***	-0.360***
			(0.000)	(0.000)	(0.000)	(0.000)
Education			0.201***	0.201***	0.0985***	0.0850**
			(0.000)	(0.000)	(0.007)	(0.024)
In full or part time employment			-0.129	-0.129	-0.211*	-0.228**
			(0.328)	(0.328)	(0.061)	(0.045)
Student			0.605***	0.605***	0.197	0.182
			(0.001)	(0.001)	(0.238)	(0.279)
Republican			-1.076***	-1.076***	-0.540***	-0.551***
			(0.000)	(0.000)	(0.001)	(0.000)
Trust in Federal Government					0.0899***	0.0894***
					(0.003)	(0.003)
Trust in State Government					-0.0315	-0.0327
					(0.210)	(0.192)
Trust in CDC					0.490***	0.491***
					(0.000)	(0.000)
Trust in Pharmaceutical companies					0.0382	0.0381
					(0.155)	(0.156)
Trust in Tech companies					0.0106	0.00843
					(0.689)	(0.751)
Frequency of travel						0.0659
						(0.125)
Constant	6.378***	6.378***	6.252***	6.252***	3.103***	3.091***
	(0.000)	(0.000)	(0.000)	(0.000)	(0.000)	(0.000)
Observations	3942	3942	3756	3756	3690	3690
Adjusted R^2	0.003	0.003	0.174	0.174	0.381	0.381

p-values in parentheses
* $p < 0.10$, ** $p < 0.05$, *** $p < 0.01$

3. Post-hoc tests: treatments vs control

```
Pairwise comparisons of means with equal variances

over         : group_no
```

	Number of Comparisons
group_no	**3**

pass_importance	Contrast	Std. Err.	Dunnett t	Dunnett P>|t|	Dunnett [95% Conf.	Interval]
group_no						
Status Quo vs Control	**.4780176**	**.1498593**	**3.19**	**0.004**	**.1259988**	**.8300364**
Peer Effect vs Control	**.4488686**	**.1500478**	**2.99**	**0.008**	**.0964072**	**.80133**
PE + SQ vs Control	**.6649899**	**.1499722**	**4.43**	**0.000**	**.312706**	**1.017274**

```
. pwmean pass_preventspread, over(group_no) mcompare(dunnett) effects

Pairwise comparisons of means with equal variances

over         : group_no
```

	Number of Comparisons
group_no	**3**

pass_preventspread	Contrast	Std. Err.	Dunnett t	Dunnett P>|t|	Dunnett [95% Conf.	Interval]
group_no						
Status Quo vs Control	**.32639**	**.1521134**	**2.15**	**0.082**	**-.0309235**	**.6837036**
Peer Effect vs Control	**.4444529**	**.1523045**	**2.92**	**0.010**	**.0866903**	**.8022155**
PE + SQ vs Control	**.7871948**	**.1523045**	**5.17**	**0.000**	**.4294323**	**1.144957**

```
. pwmean pass_normalcy, over(group_no) mcompare(dunnett) effects

Pairwise comparisons of means with equal variances

over         : group_no
```

	Number of Comparisons
group_no	3

pass_normalcy	Contrast	Std. Err.	Dunnett t	Dunnett P>|t|	Dunnett [95% Conf.	Interval]
group_no						
Status Quo vs Control	.4092068	.1514514	2.70	0.019	.0534481	.7649654
Peer Effect vs Control	.395241	.1514898	2.61	0.025	.0393922	.7510898
PE + SQ vs Control	.737457	.1513749	4.87	0.000	.381878	1.093036

```
. pwmean pass_limitliberty, over(group_no) mcompare(dunnett) effects

Pairwise comparisons of means with equal variances

over         : group_no
```

	Number of Comparisons
group_no	3

pass_limitliberty	Contrast	Std. Err.	Dunnett t	Dunnett P>|t|	Dunnett [95% Conf.	Interval]
group_no						
Status Quo vs Control	-.3721272	.1554656	-2.39	0.045	-.7373151	-.0069392
Peer Effect vs Control	.0294156	.1555052	0.19	0.995	-.3358655	.3946967
PE + SQ vs Control	-.3833839	.1563135	-2.45	0.038	-.7505634	-.0162043

```
. pwmean pass_socialfabric, over(group_no) mcompare(dunnett) effects

Pairwise comparisons of means with equal variances

over         : group_no
```

	Number of Comparisons
group_no	3

pass_socialfabric	Contrast	Std. Err.	Dunnett t	Dunnett P>|t|	Dunnett [95% Conf.	Interval]
group_no						
Status Quo vs Control	-.2797201	.1503161	-1.86	0.155	-.632812	.0733717
Peer Effect vs Control	-.0688592	.1502775	-0.46	0.941	-.4218602	.2841418
PE + SQ vs Control	-.4682747	.1507854	-3.11	0.005	-.8224688	-.1140806

```
. pwmean pass_privacy, over(group_no) mcompare(dunnett) effects

Pairwise comparisons of means with equal variances

over         : group_no
```

	Number of Comparisons
group_no	3

pass_privacy	Contrast	Std. Err.	Dunnett t	Dunnett P>|t|	Dunnett [95% Conf.	Interval]
group_no						
Status Quo vs Control	-.3241938	.1523358	-2.13	0.086	-.6820299	.0336423
Peer Effect vs Control	-.0403011	.1520241	-0.27	0.987	-.397405	.3168029
PE + SQ vs Control	-.3484439	.1527718	-2.28	0.059	-.7073042	.0104163

```
. pwmean pass_unfair_travel, over(group_no) mcompare(dunnett) effects

Pairwise comparisons of means with equal variances

over         : group_no
```

	Number of Comparisons
group_no	3

pass_unfair_travel	Contrast	Std. Err.	Dunnett t	P>|t|	Dunnett [95% Conf.	Interval]
group_no						
Status Quo vs Control	-.2232949	.1530898	-1.46	0.328	-.5829021	.1363123
Peer Effect vs Control	.2153462	.1532478	1.41	0.358	-.1446321	.5753245
PE + SQ vs Control	-.0301113	.1537692	-0.20	0.995	-.3913145	.3310918

```
. pwmean pass_unfair_harmlocals, over(group_no) mcompare(dunnett) effects

Pairwise comparisons of means with equal variances

over         : group_no
```

	Number of Comparisons
group_no	3

pass_unfair_harmlocals	Contrast	Std. Err.	Dunnett t	P>|t|	Dunnett [95% Conf.	Interval]
group_no						
Status Quo vs Control	-.1371456	.1448321	-0.95	0.662	-.4773555	.2030643
Peer Effect vs Control	.2254693	.1448321	1.56	0.277	-.1147405	.5656792
PE + SQ vs Control	-.1923754	.1451307	-1.33	0.405	-.5332868	.1485359

```
. pwmean pass_forge, over(group_no) mcompare(dunnett) effects

Pairwise comparisons of means with equal variances

over         : group_no
```

	Number of Comparisons
group_no	3

pass_forge	Contrast	Std. Err.	Dunnett t	Dunnett P>|t|	Dunnett [95% Conf. Interval]	
group_no						
Status Quo vs Control	-.0785678	.1329817	-0.59	0.885	-.3909411	.2338055
Peer Effect vs Control	.0028756	.1330152	0.02	1.000	-.3095765	.3153276
PE + SQ vs Control	-.2136879	.1331498	-1.60	0.254	-.5264562	.0990804

```
. pwmean pass_inducevaccine, over(group_no) mcompare(dunnett) effects

Pairwise comparisons of means with equal variances

over         : group_no
```

	Number of Comparisons
group_no	3

pass_inducevaccine	Contrast	Std. Err.	Dunnett t	Dunnett P>|t|	Dunnett [95% Conf. Interval]	
group_no						
Status Quo vs Control	.0906344	.1259279	0.72	0.813	-.2051696	.3864384
Peer Effect vs Control	.0172356	.1259596	0.14	0.998	-.278643	.3131141
PE + SQ vs Control	.1935194	.1260552	1.54	0.288	-.1025835	.4896224

```
. pwmean pass_flightsinter, over(group_no) mcompare(dunnett) effects
```

Pairwise comparisons of means with equal variances

over : **group_no**

	Number of Comparisons
group_no	**3**

pass_flightsinter	Contrast	Std. Err.	Dunnett t	Dunnett P>|t|	Dunnett [95% Conf.	Interval]
group_no						
Status Quo vs Control	**.2059544**	**.1581997**	**1.30**	**0.420**	**-.1656558**	**.5775646**
Peer Effect vs Control	**.2508196**	**.1584809**	**1.58**	**0.265**	**-.1214513**	**.6230904**
PE + SQ vs Control	**.3565106**	**.1584809**	**2.25**	**0.064**	**-.0157602**	**.7287815**

```
. pwmean pass_intentionalinfection, over(group_no) mcompare(dunnett) effects
```

Pairwise comparisons of means with equal variances

over : **group_no**

	Number of Comparisons
group_no	**3**

pass_intentionalinfec~n	Contrast	Std. Err.	Dunnett t	Dunnett P>|t|	Dunnett [95% Conf.	Interval]
group_no						
Status Quo vs Control	**.0168776**	**.0753055**	**0.22**	**0.992**	**-.1600146**	**.1937698**
Peer Effect vs Control	**-.0283784**	**.0753453**	**-0.38**	**0.965**	**-.2053641**	**.1486072**
PE + SQ vs Control	**-.0196794**	**.0754253**	**-0.26**	**0.988**	**-.196853**	**.1574943**

```
. pwmean pass_proscons, over(group_no) mcompare(dunnett) effects

Pairwise comparisons of means with equal variances

over         : group_no
```

	Number of Comparisons
group_no	3

pass_proscons	Contrast	Std. Err.	Dunnett t	P>|t|	Dunnett [95% Conf.	Interval]
group_no						
Status Quo vs Control	.4123174	.1497878	2.75	0.016	.0604666	.7641681
Peer Effect vs Control	.1463275	.1497878	0.98	0.641	-.2055232	.4981782
PE + SQ vs Control	.5137298	.1498259	3.43	0.002	.1617895	.8656701

```
. pwmean f1, over(group_no) mcompare(dunnett) effects

Pairwise comparisons of means with equal variances

over         : group_no
```

	Number of Comparisons
group_no	3

f1	Contrast	Std. Err.	Dunnett t	P>|t|	Dunnett [95% Conf.	Interval]
group_no						
Status Quo vs Control	.1188021	.0460448	2.58	0.027	.0106432	.226961
Peer Effect vs Control	.053496	.0459561	1.16	0.510	-.0544547	.1614467
PE + SQ vs Control	.1414529	.0460959	3.07	0.006	.0331738	.2497321

```
. pwmean pass_importance, over(treatments) mcompare(dunnett) effects

Pairwise comparisons of means with equal variances

over         : treatments
```

	Number of Comparisons
treatments	2

pass_importance	Contrast	Std. Err.	Dunnett t	P>|t|	Dunnett [95% Conf.	Interval]
treatments						
Status Quo vs PE + SQ	-.1869724	.1500105	-1.25	0.353	-.5188154	.1448706
Peer Effect vs PE + SQ	-.2161213	.1501992	-1.44	0.257	-.5483816	.1161389

```
. pwmean pass_preventspread, over(treatments) mcompare(dunnett) effects

Pairwise comparisons of means with equal variances

over         : treatments
```

	Number of Comparisons
treatments	2

pass_preventspread	Contrast	Std. Err.	Dunnett t	P>|t|	Dunnett [95% Conf.	Interval]
treatments						
Status Quo vs PE + SQ	-.4608048	.1522443	-3.03	0.005	-.7975893	-.1240203
Peer Effect vs PE + SQ	-.3427419	.1524356	-2.25	0.046	-.6799495	-.0055344

```
. pwmean pass_normalcy, over(treatments) mcompare(dunnett) effects
```

Pairwise comparisons of means with equal variances

over : **treatments**

	Number of Comparisons
treatments	2

pass_normalcy	Contrast	Std. Err.	Dunnett t	P>|t|	Dunnett [95% Conf.	Interval]
treatments						
Status Quo vs PE + SQ	-.3282502	.1511233	-2.17	0.055	-.6625547	.0060542
Peer Effect vs PE + SQ	-.342216	.1511615	-2.26	0.044	-.676605	-.0078269

```
. pwmean pass_limitliberty, over(treatments) mcompare(dunnett) effects
```

Pairwise comparisons of means with equal variances

over : **treatments**

	Number of Comparisons
treatments	2

pass_limitliberty	Contrast	Std. Err.	Dunnett t	P>|t|	Dunnett [95% Conf.	Interval]
treatments						
Status Quo vs PE + SQ	.0112567	.1564566	0.07	0.996	-.3348457	.3573592
Peer Effect vs PE + SQ	.4127995	.156496	2.64	0.016	.0666098	.7589892

```
. pwmean pass_socialfabric, over(treatments) mcompare(dunnett) effects

Pairwise comparisons of means with equal variances

over         : treatments
```

	Number of Comparisons
treatments	2

pass_socialfabric	Contrast	Std. Err.	Dunnett t	P>|t|	Dunnett [95% Conf.	Interval]
treatments						
Status Quo vs PE + SQ	.1885545	.1504126	1.25	0.349	−.144178	.521287
Peer Effect vs PE + SQ	.3994155	.1503742	2.66	0.015	.0667679	.732063

```
. pwmean pass_privacy, over(treatments) mcompare(dunnett) effects

Pairwise comparisons of means with equal variances

over         : treatments
```

	Number of Comparisons
treatments	2

pass_privacy	Contrast	Std. Err.	Dunnett t	P>|t|	Dunnett [95% Conf.	Interval]
treatments						
Status Quo vs PE + SQ	.0242501	.1535262	0.16	0.982	−.3153699	.3638702
Peer Effect vs PE + SQ	.3081429	.153214	2.01	0.081	−.0307866	.6470724

```
. pwmean pass_unfair_travel, over(treatments) mcompare(dunnett) effects

Pairwise comparisons of means with equal variances

over         : treatments
```

	Number of Comparisons
treatments	2

pass_unfair_travel	Contrast	Std. Err.	Dunnett t	Dunnett P>|t|	Dunnett [95% Conf.	Interval]
treatments						
Status Quo vs PE + SQ	-.1931835	.1536769	-1.26	0.347	-.533137	.1467699
Peer Effect vs PE + SQ	.2454575	.153834	1.60	0.193	-.0948435	.5857585

```
. pwmean pass_unfair_harmlocals, over(treatments) mcompare(dunnett) effects

Pairwise comparisons of means with equal variances

over         : treatments
```

	Number of Comparisons
treatments	2

pass_unfair_harmlocals	Contrast	Std. Err.	Dunnett t	Dunnett P>|t|	Dunnett [95% Conf.	Interval]
treatments						
Status Quo vs PE + SQ	.0552299	.1458728	0.38	0.901	-.26746	.3779197
Peer Effect vs PE + SQ	.4178448	.1458728	2.86	0.008	.0951549	.7405346

```
. pwmean pass_forge, over(treatments) mcompare(dunnett) effects

Pairwise comparisons of means with equal variances

over         : treatments
```

	Number of Comparisons
treatments	2

pass_forge	Contrast	Std. Err.	Dunnett t	Dunnett P>|t|	Dunnett [95% Conf. Interval]	
treatments						
Status Quo vs PE + SQ	.1351201	.132768	1.02	0.490	-.1585802	.4288204
Peer Effect vs PE + SQ	.2165635	.1328014	1.63	0.180	-.0772106	.5103375

```
. pwmean pass_inducevaccine, over(treatments) mcompare(dunnett) effects

Pairwise comparisons of means with equal variances

over         : treatments
```

	Number of Comparisons
treatments	2

pass_inducevaccine	Contrast	Std. Err.	Dunnett t	Dunnett P>|t|	Dunnett [95% Conf. Interval]	
treatments						
Status Quo vs PE + SQ	-.102885	.1267562	-0.81	0.629	-.3832863	.1775163
Peer Effect vs PE + SQ	-.1762839	.1267881	-1.39	0.279	-.4567557	.1041879

. pwmean pass_flightsinter, over(treatments) mcompare(dunnett) effects

Pairwise comparisons of means with equal variances

over : **treatments**

	Number of Comparisons
treatments	**2**

pass_flightsinter	Contrast	Std. Err.	Dunnett t	Dunnett P>|t|	Dunnett [95% Conf.	Interval]
treatments						
Status Quo vs PE + SQ	**-.1505562**	**.1585631**	**-0.95**	**0.535**	**-.5013186**	**.2002061**
Peer Effect vs PE + SQ	**-.1056911**	**.1588438**	**-0.67**	**0.730**	**-.4570745**	**.2456924**

. pwmean pass_intentionalinfection, over(treatments) mcompare(dunnett) effects

Pairwise comparisons of means with equal variances

over : **treatments**

	Number of Comparisons
treatments	**2**

pass_intentionalinfec~n	Contrast	Std. Err.	Dunnett t	Dunnett P>|t|	Dunnett [95% Conf.	Interval]
treatments						
Status Quo vs PE + SQ	**.036557**	**.0747995**	**0.49**	**0.842**	**-.1289094**	**.2020234**
Peer Effect vs PE + SQ	**-.008699**	**.0748389**	**-0.12**	**0.990**	**-.1742526**	**.1568545**

```
. pwmean pass_proscons, over(treatments) mcompare(dunnett) effects

Pairwise comparisons of means with equal variances

over         : treatments
```

	Number of Comparisons
treatments	2

pass_proscons	Contrast	Std. Err.	Dunnett t	Dunnett P>|t|	Dunnett [95% Conf.	Interval]
treatments						
Status Quo vs PE + SQ	-.1014124	.1494077	-0.68	0.721	-.4319219	.229097
Peer Effect vs PE + SQ	-.3674023	.1494077	-2.46	0.026	-.6979117	-.0368929

```
. pwmean f1, over(treatments) mcompare(dunnett) effects

Pairwise comparisons of means with equal variances

over         : treatments
```

	Number of Comparisons
treatments	2

f1	Contrast	Std. Err.	Dunnett t	Dunnett P>|t|	Dunnett [95% Conf.	Interval]
treatments						
Status Quo vs PE + SQ	-.0226508	.04662	-0.49	0.843	-.1257803	.0804786
Peer Effect vs PE + SQ	-.087957	.0465308	-1.89	0.106	-.1908892	.0149753

vaccine_status	Freq.	Percent	Cum.
Both doses received	2,277	57.07	57.07
No dose: doesn't want vaccine	368	9.22	66.29
No dose: doubts over vaccine	475	11.90	78.20
No dose: wants vaccine	410	10.28	88.47
One dose: doubts on 2nd	23	0.58	89.05
One dose: intention to get 2nd	431	10.80	99.85
One dose: no intention for 2nd	6	0.15	100.00
Total	3,990	100.00	

4. Spillover effects on vaccination intention

Table S15: Determinants of vaccination intention for respondents who have not yet received any dose of the vaccine after the introduction of the pass table

	(1)<br>Vaccination intention	(2)<br>Vaccination intention	(3)<br>Vaccination intention	(4)<br>Vaccination intention	(5)<br>Vaccination intention	(6)<br>Vaccination intention
Status Quo	0.0111	0.0111	-0.0681	-0.0681	0.00432	0.0124
	(0.928)	(0.928)	(0.546)	(0.546)	(0.964)	(0.897)
Peer Effect	-0.265**	-0.265**	-0.239**	-0.239**	-0.124	-0.120
	(0.033)	(0.033)	(0.044)	(0.044)	(0.223)	(0.238)
Peer Effect Status Quo	-0.192	-0.192	-0.178	-0.178	-0.0225	-0.0103
	(0.122)	(0.122)	(0.126)	(0.126)	(0.824)	(0.919)
Age			-0.0189***	-0.0189***	-0.0132***	-0.0123***
			(0.000)	(0.000)	(0.000)	(0.000)
Income			-0.00175	-0.00175	0.00151	-0.00233
			(0.896)	(0.896)	(0.895)	(0.840)
Female			-0.128	-0.128	-0.0798	-0.0673
			(0.129)	(0.129)	(0.270)	(0.357)
White			-0.145	-0.145	-0.111	-0.0918
			(0.130)	(0.130)	(0.183)	(0.275)
Political Scale			-0.240***	-0.240***	-0.141***	-0.138***
			(0.000)	(0.000)	(0.000)	(0.000)
Education			0.101***	0.101***	0.0925***	0.0747**
			(0.003)	(0.003)	(0.002)	(0.014)
In full or part time employment			0.00988	0.00988	-0.0611	-0.0806
			(0.918)	(0.918)	(0.482)	(0.355)
Student			0.573***	0.573***	0.498***	0.479***
			(0.000)	(0.000)	(0.001)	(0.001)
Republican			0.00900	0.00900	0.0487	0.0307
			(0.943)	(0.943)	(0.630)	(0.763)
Trust in Federal Government					0.0460*	0.0451*
					(0.094)	(0.099)
Trust in State Government					-0.0430**	-0.0431**
					(0.042)	(0.042)
Trust in CDC					0.224***	0.226***
					(0.000)	(0.000)
Trust in Pharmaceutical companies					0.0602***	0.0589***
					(0.007)	(0.008)
Trust in Tech companies					-0.0308	-0.0346*
					(0.138)	(0.098)
Frequency of travel						0.0812**
						(0.028)
Constant	3.017***	3.017***	4.246***	4.246***	2.585***	2.557***
	(0.000)	(0.000)	(0.000)	(0.000)	(0.000)	(0.000)
Observations	1253	1253	1192	1192	1158	1158
Adjusted R^2	0.004	0.004	0.170	0.170	0.400	0.403

p-values in parentheses

* $p < 0.10$, ** $p < 0.05$, *** $p < 0.01$

Table S16: Determinants of vaccination intention for respondents who already received the first dose of the vaccine after the introduction of the pass table

	(1)	(2)	(3)	(4)	(5)	(6)
	Vaccination intention	Vaccination intention	Vaccination intention	Vaccination intention	Vaccination intention	Vaccination intention
Status Quo	-0.209	-0.209	-0.247*	-0.247*	-0.271*	-0.275*
	(0.152)	(0.152)	(0.085)	(0.085)	(0.062)	(0.059)
Peer Effect	-0.0247	-0.0247	-0.0580	-0.0580	-0.0792	-0.0807
	(0.838)	(0.838)	(0.631)	(0.631)	(0.507)	(0.501)
Peer Effect Status Quo	0.196*	0.196*	0.161	0.161	0.106	0.103
	(0.055)	(0.055)	(0.106)	(0.106)	(0.290)	(0.310)
Age			0.0000167	0.0000167	0.000856	0.000271
			(0.997)	(0.997)	(0.872)	(0.959)
Income			0.0267*	0.0267*	0.0235	0.0243*
			(0.055)	(0.055)	(0.104)	(0.092)
Female			0.242***	0.242***	0.223**	0.213**
			(0.006)	(0.006)	(0.013)	(0.015)
White			0.106	0.106	0.116	0.114
			(0.233)	(0.233)	(0.202)	(0.208)
Political Scale			-0.0885***	-0.0885***	-0.0906***	-0.0905***
			(0.002)	(0.002)	(0.003)	(0.003)
Education			-0.0281	-0.0281	-0.0450	-0.0353
			(0.439)	(0.439)	(0.227)	(0.388)
In full or part time employment			-0.0621	-0.0621	-0.0679	-0.0516
			(0.552)	(0.552)	(0.550)	(0.646)
Student			0.0956	0.0956	0.0781	0.0842
			(0.490)	(0.490)	(0.593)	(0.568)
Republican			-0.219	-0.219	-0.106	-0.0954
			(0.293)	(0.293)	(0.595)	(0.629)
Trust in Federal Government					0.0288	0.0275
					(0.342)	(0.357)
Trust in State Government					-0.0334	-0.0316
					(0.238)	(0.257)
Trust in CDC					0.0307	0.0309
					(0.215)	(0.212)
Trust in Pharmaceutical companies					0.00433	0.00604
					(0.855)	(0.796)
Trust in Tech companies					0.0267	0.0272
					(0.236)	(0.229)
Frequency of travel						-0.0325
						(0.465)
Constant	4.529***	4.529***	4.583***	4.583***	4.375***	4.374***
	(0.000)	(0.000)	(0.000)	(0.000)	(0.000)	(0.000)
Observations	460	460	445	445	433	433
Adjusted R^2	0.017	0.017	0.085	0.085	0.097	0.096

p-values in parentheses
* $p < 0.10$, ** $p < 0.05$, *** $p < 0.01$

Table S17: Determinants of vaccination intention for respondents who already received both doses of the vaccine after the introduction of the pass table (if a new vaccination cycle is required)

	(1)<br>Vaccination intention	(2)<br>Vaccination intention	(3)<br>Vaccination intention	(4)<br>Vaccination intention	(5)<br>Vaccination intention	(6)<br>Vaccination intention
Status Quo	-0.0523	-0.0523	-0.0184	-0.0184	-0.0302	-0.0304
	(0.263)	(0.263)	(0.693)	(0.693)	(0.497)	(0.494)
Peer Effect	0.000108	0.000108	0.00559	0.00559	0.00849	0.00696
	(0.998)	(0.998)	(0.905)	(0.905)	(0.852)	(0.879)
Peer Effect Status Quo	-0.0504	-0.0504	-0.0176	-0.0176	-0.0231	-0.0258
	(0.291)	(0.291)	(0.714)	(0.714)	(0.613)	(0.572)
Age			0.000666	0.000666	-0.00100	-0.000842
			(0.681)	(0.681)	(0.534)	(0.599)
Income			0.0169***	0.0169***	0.0107**	0.00955*
			(0.001)	(0.001)	(0.033)	(0.062)
Female			0.0438	0.0438	0.0531	0.0536
			(0.208)	(0.208)	(0.117)	(0.114)
White			0.0177	0.0177	0.00951	0.0133
			(0.663)	(0.663)	(0.811)	(0.740)
Political Scale			-0.0871***	-0.0871***	-0.0749***	-0.0747***
			(0.000)	(0.000)	(0.000)	(0.000)
Education			0.0145	0.0145	0.0154	0.0126
			(0.315)	(0.315)	(0.268)	(0.378)
In full or part time employment			-0.0805*	-0.0805*	-0.0841**	-0.0876**
			(0.066)	(0.066)	(0.044)	(0.036)
Student			-0.0323	-0.0323	-0.0828	-0.0864
			(0.621)	(0.621)	(0.191)	(0.173)
Republican			-0.152*	-0.152*	-0.0765	-0.0799
			(0.058)	(0.058)	(0.316)	(0.297)
Trust in Federal Government					0.0137	0.0133
					(0.226)	(0.237)
Trust in State Government					-0.0116	-0.0119
					(0.221)	(0.209)
Trust in CDC					0.0686***	0.0691***
					(0.000)	(0.000)
Trust in Pharmaceutical companies					0.0140	0.0143
					(0.172)	(0.161)
Trust in Tech companies					0.00523	0.00482
					(0.647)	(0.671)
Frequency of travel						0.0182
						(0.254)
Constant	4.706***	4.706***	4.717***	4.717***	4.241***	4.235***
	(0.000)	(0.000)	(0.000)	(0.000)	(0.000)	(0.000)
Observations	2275	2275	2158	2158	2121	2121
Adjusted R^2	-0.000	-0.000	0.055	0.055	0.115	0.115

p-values in parentheses
* $p < 0.10$, ** $p < 0.05$, *** $p < 0.01$

Table S18: Determinants of vaccination intention after the introduction of the pass table (aggregating all vaccination intention answers)

	(1)<br>Vaccination intention	(2)<br>Vaccination intention	(3)<br>Vaccination intention	(4)<br>Vaccination intention	(5)<br>Vaccination intention	(6)<br>Vaccination intention
Status Quo	-0.0942	-0.0942	-0.0692	-0.0692	-0.0553	-0.0535
	(0.115)	(0.115)	(0.213)	(0.213)	(0.262)	(0.279)
Peer Effect	-0.126**	-0.126**	-0.107*	-0.107*	-0.0695	-0.0705
	(0.039)	(0.039)	(0.062)	(0.062)	(0.175)	(0.169)
Peer Effect Status Quo	-0.0978	-0.0978	-0.0501	-0.0501	-0.0318	-0.0331
	(0.106)	(0.106)	(0.368)	(0.368)	(0.522)	(0.505)
Age			-0.0000774	-0.0000774	-0.00176	-0.00126
			(0.968)	(0.968)	(0.320)	(0.475)
Income			0.0341***	0.0341***	0.0213***	0.0186***
			(0.000)	(0.000)	(0.000)	(0.001)
Female			-0.0743*	-0.0743*	-0.0312	-0.0258
			(0.071)	(0.071)	(0.399)	(0.486)
White			0.0115	0.0115	-0.00302	0.00627
			(0.805)	(0.805)	(0.943)	(0.882)
Political Scale			-0.229***	-0.229***	-0.159***	-0.158***
			(0.000)	(0.000)	(0.000)	(0.000)
Education			0.161***	0.161***	0.120***	0.111***
			(0.000)	(0.000)	(0.000)	(0.000)
In full or part time employment			-0.0167	-0.0167	-0.0318	-0.0437
			(0.762)	(0.762)	(0.520)	(0.376)
Student			0.449***	0.449***	0.282***	0.271***
			(0.000)	(0.000)	(0.000)	(0.000)
Republican			-0.339***	-0.339***	-0.155**	-0.163**
			(0.000)	(0.000)	(0.031)	(0.023)
Trust in Federal Government					0.0305**	0.0301**
					(0.013)	(0.014)
Trust in State Government					-0.0257***	-0.0266***
					(0.010)	(0.008)
Trust in CDC					0.178***	0.179***
					(0.000)	(0.000)
Trust in Pharmaceutical companies					0.0600***	0.0599***
					(0.000)	(0.000)
Trust in Tech companies					-0.0310***	-0.0324***
					(0.008)	(0.005)
Frequency of travel						0.0474***
						(0.008)
Constant	4.181***	4.181***	3.910***	3.910***	2.827***	2.818***
	(0.000)	(0.000)	(0.000)	(0.000)	(0.000)	(0.000)
Observations	3993	3993	3799	3799	3716	3716
Adjusted R^2	0.000	0.000	0.195	0.195	0.357	0.358

p-values in parentheses
* $p < 0.10$, ** $p < 0.05$, *** $p < 0.01$
